# Solvent-particles interactions during composite particles formation by pulsed laser melting of α-Fe_2_O_3_

**DOI:** 10.1038/s41598-022-15729-y

**Published:** 2022-07-13

**Authors:** M. S. Shakeri, O. Polit, B. Grabowska-Polanowska, A. Pyatenko, K. Suchanek, M. Dulski, J. Gurgul, Z. Swiatkowska-Warkocka

**Affiliations:** 1 Institute of Nuclear Physics Polish Academy of Sciences, PL-31342 Krakow, Poland; 2 Institute of Technology and Life Sciences-National Research Institute, Al. Hrabska 3, 05-090 Raszyn, Poland; 3 The National Institute of Advanced Industrial Science and Technology (AIST), Tsukuba, Ibaraki 305-8560 Japan; 4 Department of Physics, Cracow University of Technology, Podchorążych 1, 30-084 Kraków, Poland; 5 University of Silesia, 40-007 Katowice, Poland; 6 Jerzy Haber Institute of Catalysis and Surface Chemistry Polish Academy of Sciences, Niezapominajek 8, 30-239 Krakow, Poland

**Keywords:** Composites, Synthesis and processing, Structural properties, Theory and computation

## Abstract

This work thoroughly investigates chemical solvent-particles interactions during the formation of composite particles by pulsed laser melting of α-Fe_2_O_3_. Two solvents, with different dielectric constants, such as ethyl acetate (ε_r_ = 6) and ethanol (ε_r_ = 24.6), were examined in terms of their effect on the morphology, size, and phase composition of iron oxide composites. We calculated the laser fluence curves using the heating-melting-evaporation approach to identify the critical particle size that undergoes the phase changes first. We assessed the temperature of the particles irradiated with 390 mJ/pulse^.^cm^2^ in both solvents, including the heat dissipation between the particles and the liquid. The phase diagram of the Fe–O–C–H system was calculated to determine the temperature–pressure relationship of the system in equilibrium. We also employed an in situ GC–MS analysis to identify the volatile products during irradiation. Based on our experimental results, we concluded that the final diameter of the composites increases from 400 to 600 nm, along with the decreasing dielectric constant of the solvent, which is related to the different polarization of the organic liquid and the degree of particle agglomeration. The reduction of hematite in ethanol proceeded much faster, ending up with Fe/FeC_x_, while in ethyl acetate, it ended up with Fe_3_O_4_. Among all the particles, those with a diameter of 200 nm have the highest temperature and undergo the phase transition first. The temperature of a 200 nm composite particle in ethanol is slightly lower than in ethyl acetate, i.e. 1870 K as compared to 1902 K. Phase equilibrium diagrams proved the existence of Fe, FeO, and Fe_3_O_4_ as the preferred phases at about 1900 K. Our research provides a new insight into the process of submicron particle formation during pulsed laser irradiation and allows proposing a mechanism for the growth of particles of different size and phase composition depending on the solvent.

## Introduction

In the last decades, laser processing has become an important route for synthesizing nanoparticles^1^. The pulsed laser ablation in liquid (LAL) method uses a focused laser beam that can provide high energy density to small areas on the target leading to rapid growth of explosive nanoparticles of pure metals^2,3^, metallic glasses^4^, bimetallic particles with various architectures like alloys^5–13^, or core–shell^14^). Using an unfocused laser beam of moderate fluence for irradiation of nanoparticles dispersed in liquid medium results in a slightly different phenomenon. The irradiated material melts and subsequently merges to form submicrometer-sized spherical particles^15–17^. The latter technique referred to as pulsed laser melting in liquids (LML) proved to be a comprehensive and promising method for the synthesis of colloidal submicrometer spheres with outstanding properties. These particles with fascinating characteristics have a great potential in biosensing, medical application, energy storage, catalysis, photonics, and many others^18–21^.

So far, the LML method has shown to be an effective approach to the synthesis of composite particles (i.e. particles that consist of two or more constituent materials) with different morphology, i.e. core-shells and alloys, as well as different phase compositions, i.e. metals, oxides, or non-equilibrium bimetallic alloys (AuFe, AuCo, and AuNi). Size, morphology, and composition of obtained particles can be adjusted in a controllable manner by experimental parameters, such as wavelength^22^, solvent^23,24^, concentration^25^, irradiation time^24,26^, laser fluence^27–33^, or molar ratio of irradiated materials^33^. It has been reported that laser irradiation of colloid suspensions in organic solvents leads to a decrease in the oxidation state of irradiated oxides^16,17,29–34^. For example, a reduction of copper oxide (CuO) to the metallic phase occurs only in an organic solvent like ethanol or acetonitrile, whereas it does not take place in water^17^. In addition, it has been reported that during pulsed laser melting, reducing gases such as carbon monoxide and hydrocarbons are formed around the particles due to the high pressure and temperature conditions^34,35^. Other parameters like photolysis, thermal decomposition of the solvent, and the interaction of the chemical compounds resulting from pyrolysis with the particle surface can perform a significant role in creating composite particles during the pulse laser irradiation process. Although the gases produced during the reduction process may be important in reducing the oxidation state of irradiated oxides, the radical reactions that occur during the laser processes have not been extensively investigated so far^34,36,37^. For instance, the reduction of Fe_3_O_4_ in ethanol during laser melting is explained by the evaporation of ethanol surrounding the particles and the formation of ethylene compounds as reducing agents. Suehara et al., using computational simulations, showed that ethylene is the main product of ethanol decomposition during pulsed laser heating for 100 ns at 1000–4000 K^34^. In another paper, a partial reduction of bismuth oxide to metallic Bi during pulsed laser irradiation was explained by the interaction of Bi-oxides with H_2_ and CO molecules obtained by the decomposition of ethanol^36^. The interactions of water and ethanol with the titania surface were investigated by spectroscopic characterizations and ab initio calculations^37^. It was shown that both solvents interact with oxygen vacancies on the titania surface, resulting in partial passivation of defects by water and their complete passivation by ethanol molecules. Exploring the interactions between the irradiated material and solvent molecules including the investigation of the thermodynamic behavior of particles as well as the thermal modeling of the system under various circumstances is needed to improve the controllability of produced materials with specific structures and unique properties.

This work investigates for the first time how organic solvents with various dielectric constants influence the morphology and composition of particles during the LML process. Based on theoretical and experimental results, we offer an insight into the impact of two solvents (ethyl acetate and ethanol) on the synthesis of composite particles focusing on the size and phase composition. Moreover, we approach developing an analytical method for determining the gas products of the synthesis and studying its applicability for understanding the chemical processes occurring during laser irradiation of nanoparticles dispersed in a liquid.

## Results and discussion

### Role of the dielectric constant

**Figure 1:**
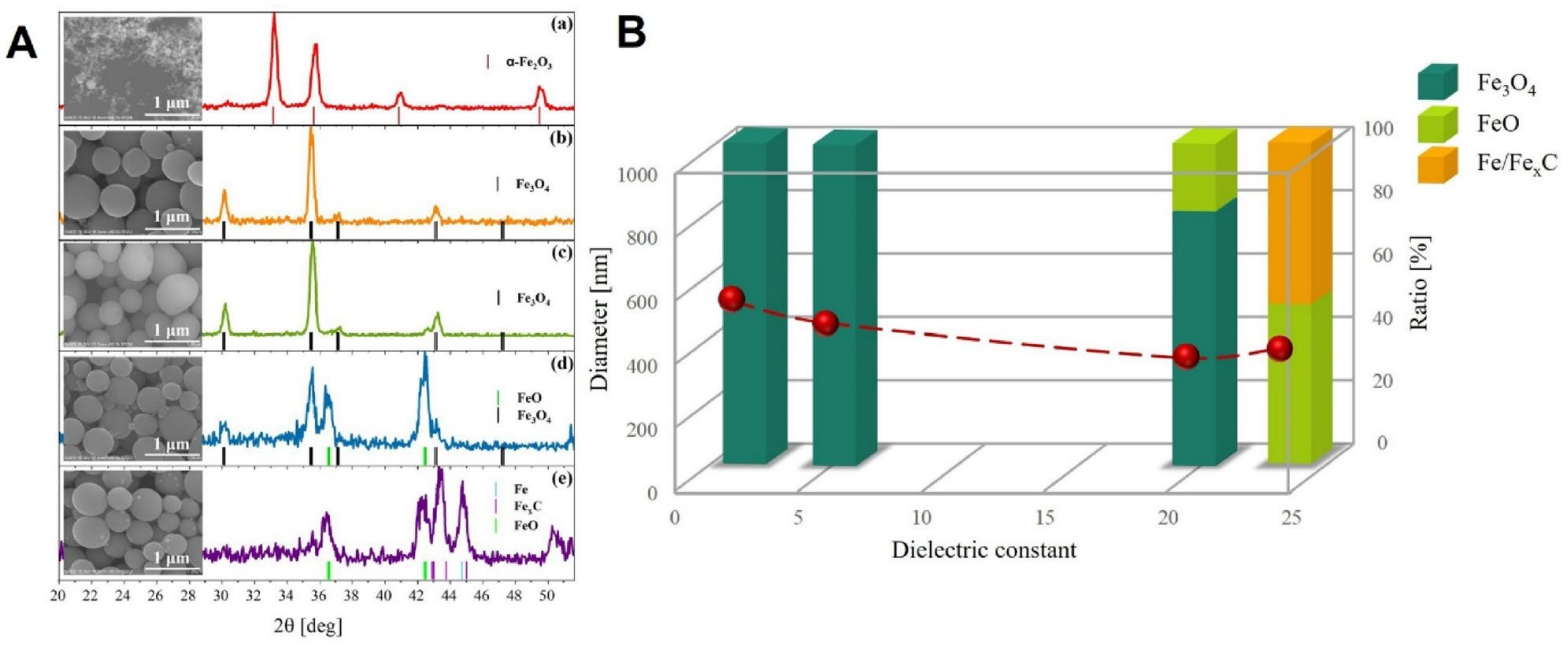
(**A**) SEM images (left side) and XRD patterns (right side) of (a) α-Fe_2_O_3_ NPs and (b–e) nanocomposites obtained by pulsed laser irradiation of α-Fe_2_O_3_ NPs in different organic solvents: (b) toluene, (c) ethyl acetate, (d) acetone, (e) ethanol. Pulsed laser irradiation with 532 nm and with 180 mJ/pulse^.^cm^2^ for 1 h, pulse frequency 30 Hz; (**B**) the final diameter of the synthesized particles is a function of the dielectric constant of the organic liquid (left axis, points). The percentage phase composition of the particles (right axis, bars).

First, to thoroughly understand the reduction of iron oxides during pulsed laser irradiation, hematite nanoparticles were irradiated in four different solvents, with different values of dielectric constants, i.e. toluene (ε_r_ = 2.4), ethyl acetate (ε_r_ = 6), acetone (ε_r_ = 20.7), and ethanol (ε_r_ = 24.6)^38^. Figure 1A shows SEM images and corresponding XRD results of raw α-Fe_2_O_3_ NPs (Fig. 1a) and particles obtained after irradiation of α-Fe_2_O_3_ NPs suspended in toluene, ethyl acetate, acetone, and ethanol solvents (Fig. 1b–e, respectively). Figure S1 shows size distributions reconstructed from SEM images of obtained particles (Supporting Information S1). In the case of raw α-Fe_2_O_3_ NPs, an agglomeration of particles is observed, while after irradiation SEM images reveal the formation of almost similar spherical microparticles. The average size of the spheres depends on the used solvent. The particles obtained in toluene and ethyl acetate are larger (approx. 600 nm in diameter) than those obtained in ethanol or acetone (approx. 400 nm in diameter). The dependence of the particle diameter on the dielectric constant of the solution is illustrated in Fig. 1B. XRD patterns shown on the right side of Fig. 1A reveal that the pulsed laser irradiation of α-Fe_2_O_3_ NPs in different solvents leads to different hematite reduction pathways, despite the same laser parameters. Only α-the Fe_2_O_3_ NPs phase (JCPDS card no. 80-2377) exists for the raw material (Fig. 1a). After irradiation in ethyl acetate and toluene, the peaks characteristic of magnetite (Fe_3_O_4_, JCPDS card no. 88–0315) are observed, as in Fig. 1b,c. After irradiation in acetone in addition to magnetite, wustite phase (FeO, JCPDS card no. 46-1312) is also present (Fig. 1d). Finally, irradiation in ethanol leads to the appearance of the peaks characteristic of iron (Fe, JCPDS card no. 85-1410) and iron carbide (Fe_x_C, JCPDS card no. 03-1056, 36-1249). Furthermore, XRD images were analyzed in detail to quantify the content of individual phases in the composites. We calculated the total area under the XRD peaks for each phase (Fe_3_O_4_, FeO, Fe/FeC_x_). The results obtained for the different liquid mediums are presented in Fig. 1B, wherein the phase composition is expressed as a function of the dielectric constant of the solvent. There is a one-step reduction path for α-Fe_2_O_3_ NPs irradiated in ethyl acetate and toluene, and a multi-step reduction path to metallic iron, and even iron carbides, for α-Fe_2_O_3_ NPs irradiated in acetone and ethanol. In addition to the SEM and XRD analyses, we also used DLS to measure the hydrodynamic diameter of raw α-Fe_2_O NPs dispersed in the discussed solvents. We found that the low dielectric constant of the medium helps to obtain large hydrodynamic diameters of the formed Fe_2_O_3_ agglomerates (Supporting Information, Table S1). The agglomerate in toluene was 800 nm, in ethyl acetate 500 nm, and in ethanol and acetone 200 nm in diameter.

Overall, these results indicate that the dielectric constant of the solvent used as the medium during pulsed laser irradiation has a great effect on both the size and the structure of the synthesized composites. Moreover, it influences the degree of agglomeration of the starting α-Fe_2_O material. The Derjaguin–Landau–Verwey–Overbeek (DLVO) theory is typically used to elucidate the phenomenon of varying particle size during pulsed laser irradiation. DLVO theory explains the aggregation of aqueous dispersions and describes the force between the charged surfaces interacting through a liquid medium^39^. Polar media with a lower value of the dielectric constant, such as toluene, have a lower value of the repulsive potential; therefore, the inter-particle repulsion is reduced leading to particle agglomeration^40^. Hence, the DLVO theory explains both the mechanism of the formation of larger particles as well as larger agglomerates of the starting material in solvents with a lower dielectric constant. Our data are also consistent with the results obtained by others authors, studying the formation of various composites using the same laser method, and stating that the dielectric constant of the solvent has an inverse effect on the size of obtained particles^15,17^. An open question, however, is why the hematite reduction pathway changes with the particle size. To explain this, the next part of this paper focuses only on two solvents, with a low and high value of the dielectric constant. We selected one representative solvent with a low dielectric constant, i.e. ethyl acetate (ε_r_ = 6), assuming that particle formation and the ongoing structural changes are similar in this group of solvents and analogously one solvent with a high dielectric constant, i.e. ethanol (ε_r_ = 24.6). For those selected experimental conditions, we conducted several additional calculations and investigations that would allow us to explain the mechanism of particle formation with a specific size and phase composition. These studies were carried out with the aid of the experimental route described in section “Characterizations” for the reasons described later on.

### Heating model

The heating model (also known as the heating − melting − evaporation (HME) model), which is based on the Mie theory, explains the formation of submicron spherical particles^22,33,41–43^. The main concept of this model is that all the energy absorbed by a particle from the laser pulse is used for the HME process. Herein, we use the HME model to compute the laser fluence curves against the particle size for specific phase transitions during the iron oxide reduction process. A description of the possible phase transitions, i.e. decomposition, melting, and evaporation with Fe_2_O_3_ as the starting material, can be found in Table 1.

**Table 1:** The thermodynamic data and chemical reactions for iron oxide phase transition with Fe_2_O_3_ as the initial material.

Name	Transformation	Temperature (K)	ΔH (kJ/kg)
J1	Decomposition of Fe_2_O_3_ (start) Fe_2_O_3_ → Fe_3_O_4_	1573	1286.964
J2	Decomposition of Fe_2_O_3_ (complete) Fe_2_O_3_ → Fe_3_O_4_	1573	1803.784
J3	Fe_3_O_4_ melting (start)	1870	1921.138
J4	Fe_3_O_4_ melting (complete)	1870	2497.561
J5	Decomposition of Fe_3_O_4_ (start) Fe_3_O_4_ → FeO	2565	3076.355
J6	Decomposition of Fe_3_O_4_ (complete) Fe_3_O_4_ → FeO	2565	4077.765
J7	FeO evaporation (start)	3687	5008.955

**Figure 2:**
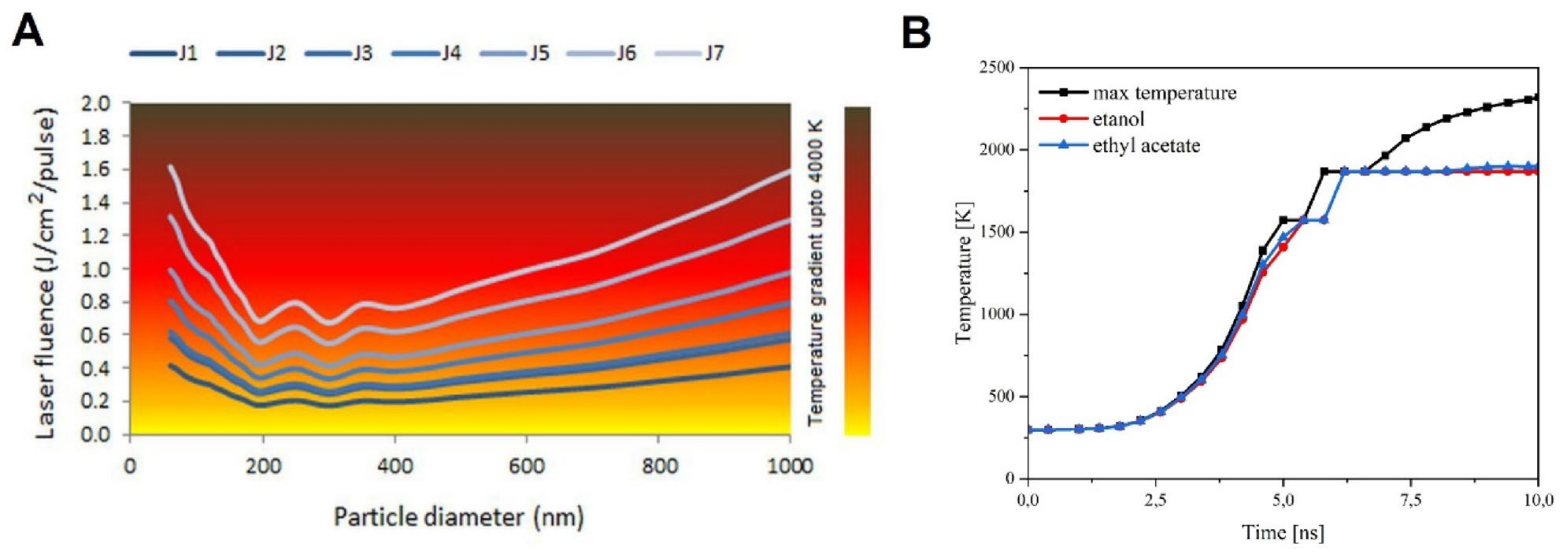
(**A**) Laser fluence (J) as a function of the particle diameter is calculated based on the HME model. Each curve J_i_, (i = 1,…7) represents a particular phase transition as indicated in Table S1; (**B**) time-dependent temperature profiles for 200 nm spherical α-Fe_2_O_3_ particles irradiated with 532 nm wavelength and with 390 mJ/pulse^.^cm^2^ laser fluence, without the cooling effect (black line), taking into account the cooling effect for ethyl acetate (blue line) and ethanol (red line).

We perform the calculations for a single pulse, assuming that the typical time needed for the processes of cooling and solidifying the particles is much longer than the duration of one pulse, and therefore any possible heat losses during the particle’s heating will be negligible. During pulsed laser irradiation, if the amount of absorbed energy is not sufficient to melt the particles, then only their heating should be expected. Increasing the laser fluence can lead to particle melting or, for a high amount of absorbed energy, to an increase in the temperature of the molten species and eventually to its evaporation. All energy absorbed by the particles could be thermodynamically expressed by

1 $${E}_{abs}=J{\sigma }_{abs}^{\lambda }\left(d\right)=\frac{{\rho }_{p }\left(\pi {d}_{p}^{3}\right)}{6}\left[\underset{{T}_{0}}{\overset{{T}_{m}}{\int }}{C}_{P}^{s}\left(T\right)dT+{\Delta H}_{m}+\underset{{T}_{m}}{\overset{T}{\int }}{C}_{P}^{l}\left(T\right)dT\right],$$

where J is the laser fluence, ${\sigma }_{abs}^{\lambda }$ is the particle absorption cross section, *d*_*p*_ is the diameter of the particle, and ${\rho }_{p}$ is the particle density, ${C}_{P}^{s}, {C}_{p}^{l}$ are the solid state and the liquid state particle heat capacity, ${T}_{0}, {T}_{m}$ are the initial and the melting temperature of the particle. According to the classical Mie theory, the value of particle absorption cross section is related to the so-called absorption efficiency as follows: ${Q}_{abs}^{\lambda }=4{\sigma }_{abs }^{\lambda }({d}_{p})/\pi {d}_{p}^{2}$. Calculated ${\sigma }_{abs}^{\lambda }$ and ${Q}_{abs}^{\lambda }$ at a wavelength of 532 nm for Fe_2_O_3_ NPs of different diameters are shown in Figure S2 (Supporting Information, Fig. S2). Using Eq. (1), which combines light absorption characteristics (Fig. S2) with thermodynamics data of iron oxides (Table 1), and taking the α-Fe_2_O_3_ density of 5.24 g/cm^3^, we determined the critical laser fluence necessary for the specific phase transition in the studied material. We assume that α-Fe_2_O_3_ NPs are irradiated with a Nd:YAG laser pulse with a wavelength of 532 nm and a pulse duration of 10 ns, and that the process takes place under adiabatic conditions, i.e. when heat is not dissipated from the particles to the surrounding liquid during pulsed heating. For molten iron oxide solutions, the boiling point is approximately 3687 K. Thus, liquid interactions under adiabatic conditions take place at temperatures below this value. The obtained results are shown in Fig. 2A, where each single curve labelled as J_i_, (i = 1,…7) determines the critical laser fluence for a particular phase transition, as indicated in Table 1, and for a given particle size. Figure 2A shows that the laser fluence has a minimum value for particles with a diameter of about 200 nm. In the case of the J_1_ curve corresponding to the beginning of Fe_2_O_3_ decomposition, it means that particles of about 200 nm in diameter can be selectively decomposed using a lower laser fluence. In other words, of all the available particle sizes, those around 200 nm will have the highest temperature. For larger or smaller ones, the same temperature is obtained with a higher value of absorbed energy.

### Heating–cooling model

As indicated above, the HME model is useful for analysing the evolution of the particle size and chemical composition but ignores the effects of particle cooling and heat transfer from the particle to the surrounding liquid. We used the HME model to determine that Fe_2_O_3_ NPs with a diameter of 200 nm will be the first to undergo the phase change as a result of laser irradiation. For this particular particle diameter, we performed further calculations taking into consideration the heat dissipation from the particle to the surrounding liquid, to derive the maximum particle temperature. We considered two liquid environments, i.e. ethyl acetate and ethanol. The relevant thermodynamic data needed for the calculations for these two solvents are provided in Table S2 (Supporting Information, Table S2). To calculate the time-dependent temperature profile of α-Fe_2_O_3_ particles with a diameter of 200 nm irradiated with Nd: YAG laser with a wavelength of 532 nm and fluence of 390 mJ/pulse^.^cm^2^, a heating–cooling model was used^41–46^. The pulse profile of an Nd: YAG laser with a pulse width of 10 ns is illustrated in Figure S3. According to the model, a conductive heat transfer can be written as

2 $$\frac{dq}{dt}=h\pi {d}_{p}^{2}\left({T}_{t}-{T}_{0}\right),$$

where πd^2^ is the particle surface area, T_t_ is the particle temperature, T_0_ is the temperature of the surrounding liquid, and *h* is the heat transfer coefficient which is defined as

3 $$h=\frac{{Nu}_{d}.K}{{d}_{p}} ,$$

where K is the heat conductivity of the surrounding liquid, Nu_d_ is the Nusselt number, and d_p_ is the particle diameter. Taking into consideration the heat transfer from the particle to the surrounding liquid, the thermal energy (E) accumulated in the particle due to the particle heating by laser beam absorption (Labs) is reduced by the factor resulting from the heat dissipation described by Eq. (2) and can be formulated according to the equation below

4 $$\frac{dE}{dt}=\frac{d{E}_{abs}}{dt}-\frac{dq}{dt} .$$

Equation (4) in combination with Eqs. (2) and (3) has an analytic solution that can describe the particle temperature below and above the melting point. If the amount of absorbed energy is too small to melt the particle, then only its heating will occur. In this case

5 $${T}_{t}(t)={T}_{0}+\frac{6{E}_{t}}{{\rho }_{p}\pi {d}^{3}{C}_{s}} .$$

Upon the attainment of the melting temperature, the particle is melting and the temperature of the droplet can be described as follows

6 $${T}_{t}(t)={T}_{0}+\frac{6{[E}_{t}-(\frac{{\rho }_{p}\pi {d}^{3}\left({\Delta H}_{{T}_{t}}+{\Delta H}_{m}\right)}{6}]}{{\rho }_{p}\pi {d}^{3}{C}_{l}} ,$$

where ${\Delta H}_{{T}_{m}}$ is the enthalpy needed for heating materials to the melting point.

Using Eqs. (5) and (6), we found a time-dependent temperature profile for 200 nm spherical α-Fe_2_O_3_ particles irradiated with 532 nm wavelength. The temperature profile for the particles dispersed in ethyl acetate and ethanol are shown in Fig. 2B, as blue and red lines, respectively. In addition, the figure shows the change in particle temperature over time, assuming that there is no energy dissipation from the particle to the surrounding liquid (black line). Examining the black line, we see a continuous increase in temperature to a maximum value of 2320 K. According to Table 1, the melting point for Fe_3_O_4_ is 1870 K, which means that the laser fluence of 390 mJ/pulse^.^cm^2^ is high enough to decompose Fe_2_O_3_ particles into Fe_3_O_4_ and then completely melt them, whether they are in ethyl acetate or in ethanol. Then, considering the cooling effect, the temperature profile flattens out in the final stage for both ethanol and ethyl acetate suspended particles, in addition, the maximum temperature is solvent dependent. For ethanol, we obtained 1870 K and for ethyl acetate 1902 K, which means that the particles suspended in the former liquid reach the melting point for Fe_2_O_3_, while those in the latter exceed it slightly. The slight difference between the maximum particle temperatures in different solvents is due to the different values of the thermal conductivity of the surrounding liquids, as shown in Table S2. Now, if particles larger than 200 nm in diameter are available, they will not reach the maximum temperature and not all of them will be decomposed. Consequently, two solid phases, Fe_2_O_3_ and Fe_3_O_4_ will be present in the organic liquid. As the DLVO model states, this happens for a liquid with a lower dielectric constant where larger agglomerates of particles are formed for the same laser fluence.

### Thermodynamic stability of the Fe–O system

**Figure 3:**
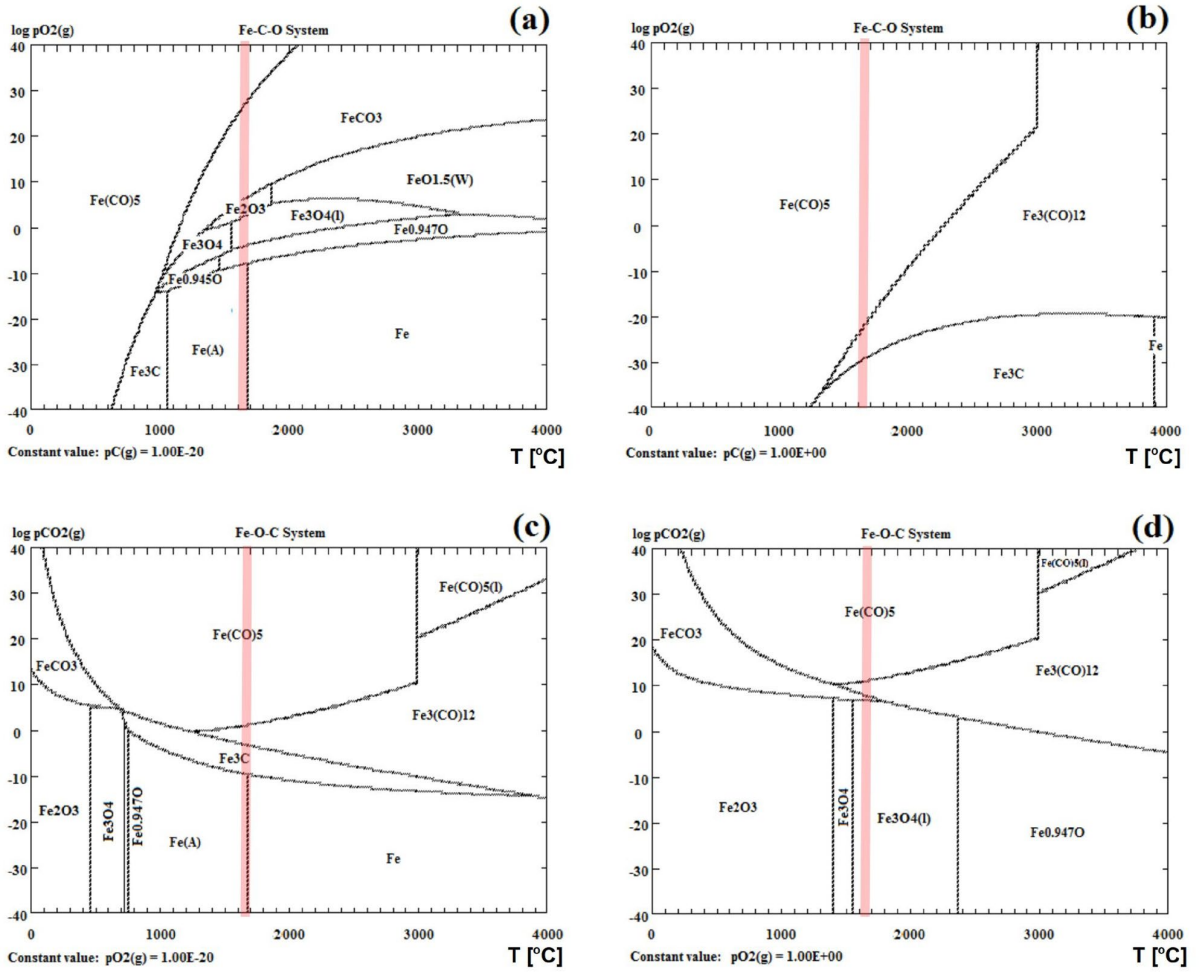
Temperature-partial pressure (TPP) phase stability diagram for ternary Fe–O-C system under (**a**,**b**) O_2_ and (c,d) CO_2_ atmospheres. (**a**) p_C_ = 10^–20^ Pa, (**b**) p_C_ = 1 Pa, (**c**) p_O2_ = 10^–20^ Pa, (**d**) p_O2_ = 1 Pa. The red vertical line indicates the maximum temperature of 200 nm particles irradiated with 390 mJ/pulse^.^cm^2^ estimated from the heating–cooling model.

**Figure 4:**
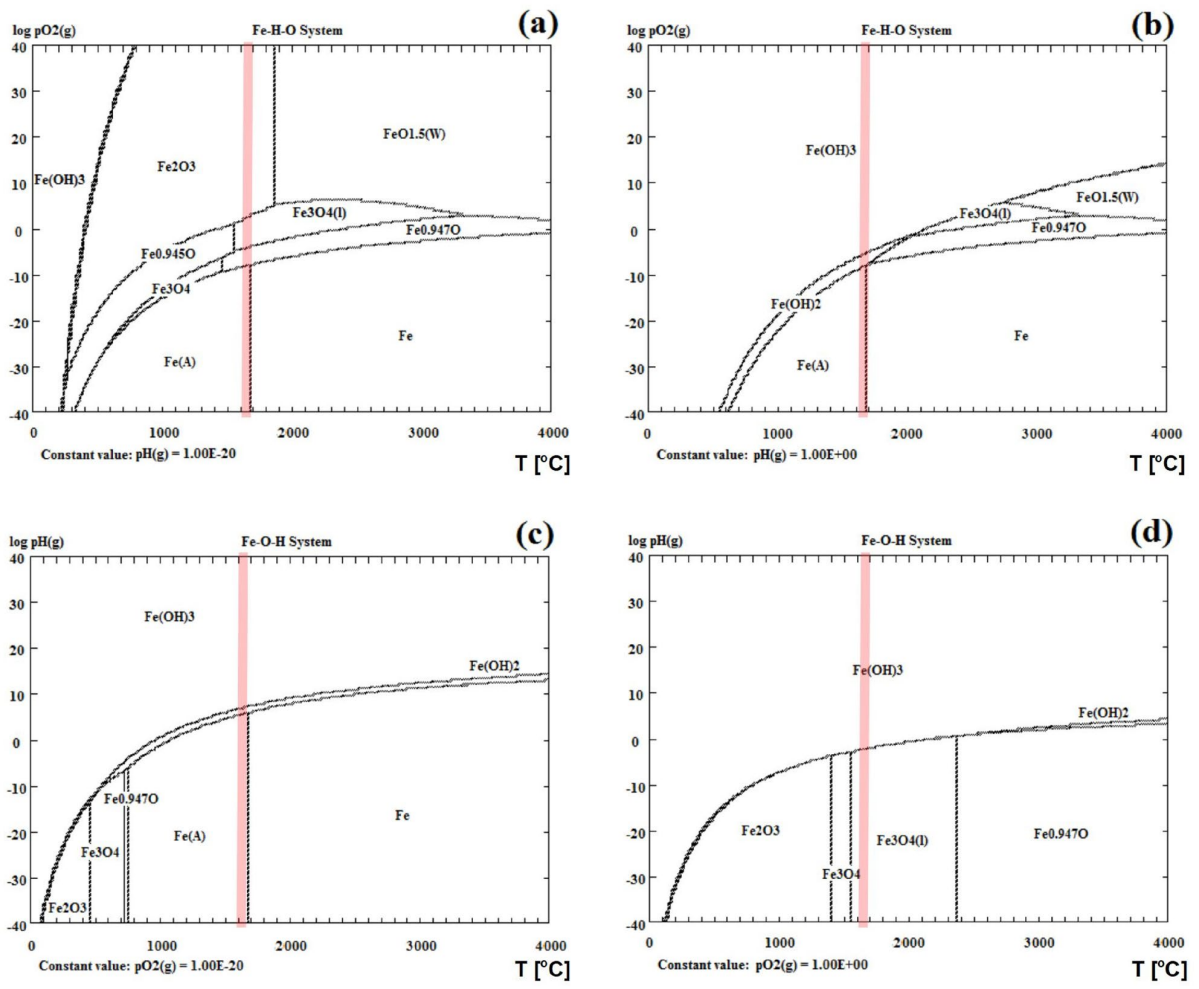
Temperature-partial pressure (TPP) phase stability diagram for the ternary Fe–O-H system under (**a**,**b**) O_2_ and (**c**,**d**) H atmospheres. (**a**) p_H_ = 10^–20^ Pa, (**b**) p_H_ = 1 Pa, (**c**) p_O2_ = 10^–20^ Pa, (**d**) p_O2_ = 1 Pa. The red vertical line indicates the maximum temperature of 200 nm particles irradiated with 390 mJ/pulse^.^cm^2^ estimated from the heating–cooling model.

For a better understanding of the possible species formation during the irradiation of the α-Fe_2_O_3_ NPs, a numerical calculation of the thermodynamic equilibrium of the—Fe–O system was carried out using hydrogen and carbon as reductants. We assumed that the temperature in the layer around the particles reaches approx. 1900 K, i.e. the maximum temperature of 200 nm particles determined from the model, as described in section “Heating–cooling model”. The calculated temperature–partial pressure (TPP) phase stability diagrams of ternary Fe–O–C and Fe–O–H systems are shown in Figs. 3 and 4, respectively.

Figure 3 shows the predominant phases of the ternary Fe–O–C system as a function of the partial pressure of oxygen (p_O2_) (Fig. 3a,b) and partial pressure of carbon dioxide (p_CO2_) (Fig. 3c,d)—in a wide range of particle temperatures. First, we performed the calculations with the assumption that the value of the partial pressure of carbon (p_C_) is minimized to about 10^–20^ Pa (Fig. 3a). Following the vertical red line indicated in Fig. 3, which defines the characteristic temperature of 200 nm particles determined from the heating–cooling model (approx. 1900 K), we can see that for low p_O2_ values, the most stable phase would be Fe. While increasing the oxygen concentrations, iron oxidizes to the stable phases of FeO, Fe_3_O_4_, and beyond 1 Pa of p_O2_ to Fe_2_O_3_. Eventually, FeCO_3_ will dominate for higher oxygen partial pressures. A similar analysis of the TPP phase diagram of the Fe–O–C system but with the assumption that the value of p_C_ = 1 Pa (Fig. 3b) shows that Fe_3_C, Fe_3_(CO)_12,_ and Fe(CO)_5_ are the most stable phases with the increasing oxygen concentration. Figure 6c,d illustrates the TPP phase stability diagram for the ternary Fe–O–C system under CO_2_ atmospheres assuming that p_O2_ = 10^–20^ Pa (Fig. 3c) and p_O2_ = 1 Pa (Fig. 3d). As seen for the former, Fe, Fe_3_C, Fe_3_(CO)_12,_ and Fe_3_(CO)_5_ are the most thermodynamically stable, whereas for the latter, Fe_3_O_4_ and Fe_3_(CO)_5_. In the case of our experimental conditions, we expected the presence of both O_2_ and CO_2_ in the solvent but due to the release of the gas from the surface of the liquid during laser irradiation, the partial pressures of both were substantially reduced, reaching the value of 1 Pa. Hence, according to our thermodynamic equilibrium calculation results (Fig. 3a,d), we can see that Fe_2_O_3_ and- Fe_3_O_4_ are the most dominant phases arising during the laser irradiation process.

In the next step, we performed numerical calculations of the thermodynamic equilibrium of the ternary Fe–O–H system due to the possible presence of hydrogen products of the organic solvents (ethyl acetate (CH_3_-COO-CH_2_-CH_3_) and ethanol (C_2_H_5_OH)) during laser irradiation. As seen in Fig. 4a,b, the preferred phases in the oxygen atmosphere at 1900 K are Fe, FeO, Fe_3_O_4_, Fe_2_O_3_, and Fe, FeO, Fe(OH)_3_ assuming low and high hydrogen pressure, respectively, that is p_H_ = 10^–20^ Pa (Fig. 4a) and p_H_ = 1 Pa (Fig. 4b). When the low value of the hydrogen pressure is set for the calculations (Fig. 4a), the phase diagram shows a similar behavior to that previously shown for the ternary Fe–O–C system in the oxygen atmosphere and for the low p_C_ = 10^–20^ Pa (Fig. 3a). The difference between these two results is relatively small, and it is visible when the p_O2_ pressure increases to about 1 Pa, where Fe_2_O_3_ and Fe_3_O_4_ are stable in the Fe–O–C system, while Fe_3_O_4_ and Fe in the Fe–O–H system. The analysis of the TPP phase diagram of the Fe–O–H system in the oxygen atmosphere, as shown in Fig. 4b, where fixed p_H_ = 1 Pa was assumed, indicates that Fe, FeO, and Fe(OH)_3_ are the most stable phases with the increasing oxygen concentration. Figure 4c,d illustrates the TPP phase stability diagram for the ternary Fe–O–H system under the H atmospheres and assuming the value of p_O2_ = 10^–20^ Pa (Fig. 4c) and p_O2_ = 1 Pa (Fig. 4d). For the former, we observed three stable phases at 2000 K, i.e. Fe, Fe(OH)_2,_ and Fe(OH)_3_, while for the latter two, i.e. Fe_3_O_4_, and Fe(OH)_3_. Overall, taking into consideration the reduced concentration of oxygen and hydrogen, due to the ongoing laser irradiation process which contributes to the release of gases from the surface of the liquid, for the Fe–O–H system Fe_2_O_3_ and Fe_3_O_4_ would be the dominant phases.

**Figure 5:**
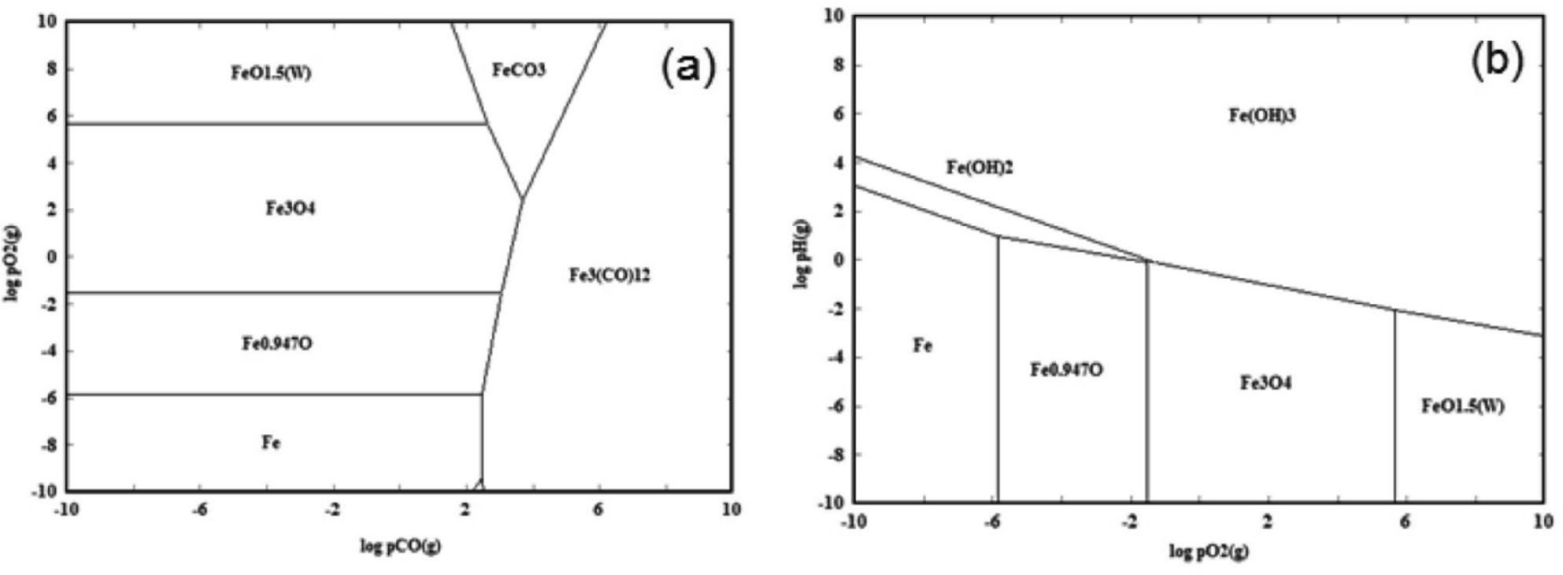
The LPP isothermal phase diagrams at 1900 K for the (**a**) Fe–O–C and (**b**) Fe–O–H systems.

Finally, we calculated the isothermal phase diagram, the so-called LPP diagram, for both considered three-component systems, i.e. for Fe–O–C (Fig. 5a) and Fe–O–H (Fig. 5b) at 2000 K. Figure 5a shows that when the pressure of oxygen and carbon dioxide is around 1 Pa, Fe_3_O_4_ will be dominant. By decreasing the p_O2_, we enter the range of FeO and Fe phase stability. Now, if we additionally take into consideration the hydrogen pressure (Fig. 5b), then again Fe_3_O_4_ will be the most stable at the oxygen concentration of 1 Pa, while below 1 Pa of p_O2_ the stable phases are FeO and Fe. Therefore, we can conclude that p_O2_ is a key factor in determining the resulting phase. The lack of oxygen or the consumption of this gas by other compounds would lead to the formationn of Fe at the end stage of the process. According to the thermodynamic calculations, while irradiating α-Fe_2_O_3_ suspended in organic solvents, Fe is the preferred phase in the F–O–C–H system at low O_2_ partial pressure, while FeO and Fe_3_O_4_ will be stable around and above 1 Pa.

### Chromatographic analysis

**Figure 6:**
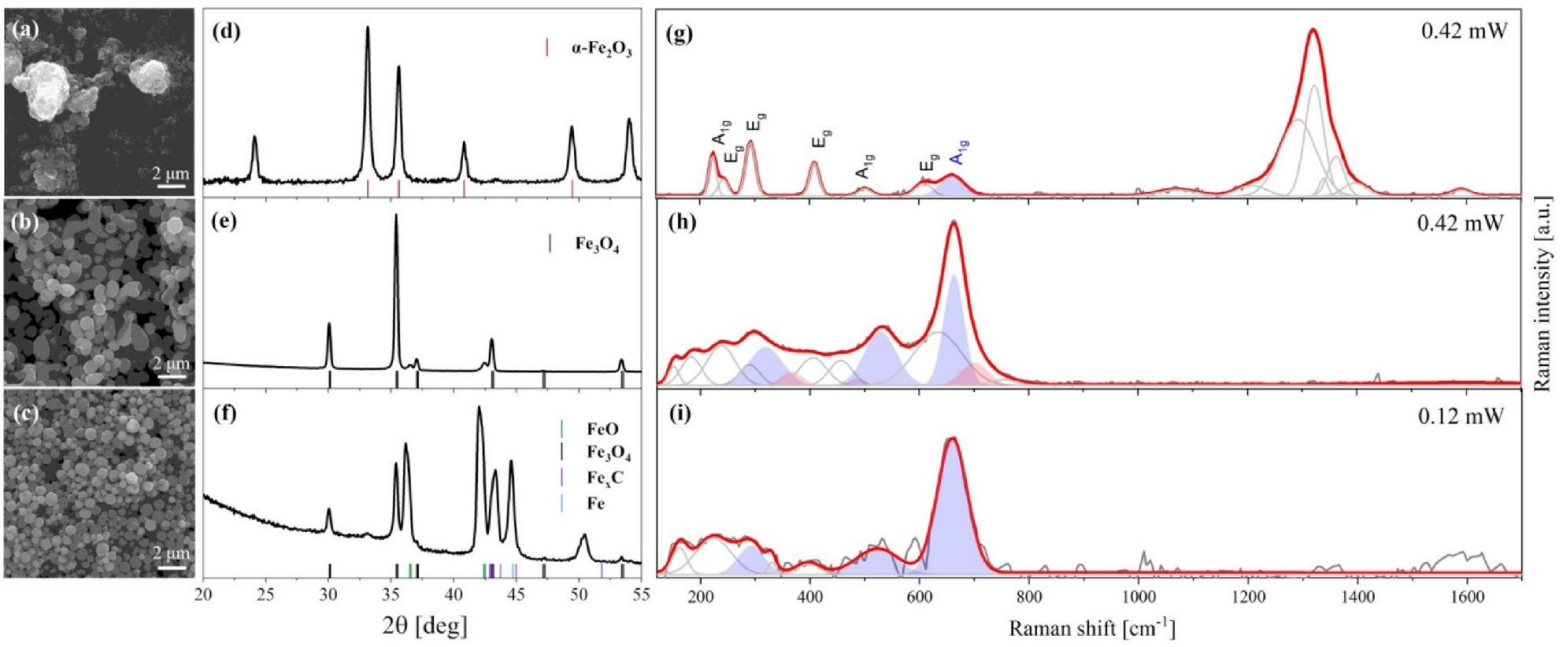
SEM images, XRD patterns and Raman spectra of α-Fe_2_O_3_ NPs and nanocomposites obtained by pulsed laser irradiation of α-Fe_2_O_3_ NPs with 532 nm and with 390 mJ/pulse^.^cm^2^ for 3 h, pulse frequency 10 Hz. (**a**,**d**,**g**) Raw α-Fe_2_O_3_ NPs, (**b**,**e**,**h**) α-Fe_2_O_3_ NPs suspended in ethyl acetate and (**c**,**f**,**i**) α-Fe_2_O_3_ NPs suspended in ethanol.

The above described theoretical calculations are the starting point for further investigation, which together will allow describing the formation process of particles with a certain size and phase composition. Apart from the characterization of the synthesized particles, we decided to trace the changes occurring in solvents as a result of photolysis during laser irradiation. Thus, the volatile products of laser irradiation of α-Fe_2_O_3_ NPs suspended in ethyl acetate and ethanol were analysed using a thermal desorption gas chromatograph and a mass spectrometer as a detector. The procedure was started with the α-Fe_2_O_3_ NPs concentration of 0.5 mM, used in the studies described in section “Role of the dielectric constant”; however, due to the low intensity of the signal, the concentration and the amount of the irradiated solution were increased to 2.5 mM and 15 ml, respectively. With the increase in the concentration of monophasic hematite nanoparticles in the solvent and the volume of the solution, the laser fluence and the irradiation time increased to 390 mJ/pulse^.^cm^2^ and 3 h, respectively. The same parameters were taken during the theoretical calculations described in the previous sections. As the irradiation conditions have changed compared to those reported in section “Role of the dielectric constant”, before the chromatographic analysis, the α-Fe_2_O_3_ nanoparticles suspended in ethyl acetate and ethanol were again examined in terms of their morphology and structure by SEM, XRD, and Raman spectroscopy, as illustrated in Fig. 6. For comparison, the results obtained for raw α-Fe_2_O_3_ NPs are also shown in Fig. 6. SEM and XRD analysis for raw NPs is analogous to that described in section “Role of the dielectric constant”. After irradiation, SEM images reveal submicron spheres of about 540 nm and 450 nm in diameter, for particles suspended in ethyl acetate (Fig. 6b) and ethanol (Fig. 6c), respectively. The results are consistent with those obtained earlier, the lower the dielectric constant of the solvent, the larger the particle size. The XRD pattern of particles formed by laser irradiation in ethyl acetate is characterized by peaks corresponding to the Fe_3_O_4_ phase (JCPDS card no. 88-0315) and the FeO phase (JCPDS card no. 46–1312) (Fig. 6e). No trace signal coming from pure α-Fe_2_O_3_ was found for this sample. The results show a reduction of hematite to magnetite and wustite. The XRD pattern of particles formed by laser irradiation in ethanol is shown in Fig. 6f. There are the peaks characteristic of the Fe_3_O_4_ and FeO phase and additionally peaks corresponding to the Fe and Fe_x_C phases (Fe, JCPDS card no. 85-1410; Fe_x_C, (JCPDS card no. 03-1056, JCPDS card no. 36-1249). The irradiation of the suspension in ethanol leads to phase transformation from hematite to the magnetite, wustite, and iron or even iron carbide phases. Here again the results are in agreement with those obtained earlier (see section “Role of the dielectric constant”), only for ethyl acetate the reduction path goes one step further. This is due to the difficulty of accurately matching the irradiation time when increasing the concentration of particles and the volume of the solution. Raman spectroscopy provides an additional insight into the structural properties of the synthesized materials (Fig. 6g–i). It was found that the raw sample (Fig. 6g) is featured by six bands centered at 608 (E_1g_), 500 (A_1g_), 408 (E_1g_), 292 (E_1g_), 244 (E_1g_), 221 (A_1g_) cm^–1^ which are assigned to the hematite phase^47^. Several bands observed around 1300 cm^–1^ are attributed to a two-magnon scattering arising from the interaction of two magnons created on antiparallel closed spins in hematite^48–50^. The high-frequency band centered at 665 cm^–1^ may originate from the presence of surface defects, reduced grain size^51–53^, or the presence of magnetite^54–56^. Different spectra were found in the case of particles obtained after laser irradiation. Raman spectra of particles formed by laser irradiation in ethyl acetate and ethanol are characterized by magnetite bands at 651 (A_1g_), 526 (T_2a_), and 321 (E_g_) cm^–1^ (Fig. 6h, blue bands), and at 658 (A_1g_), 523 (T_2a_), and 296 (E_g_) cm^–1^ (Fig. 6i, blue bands), respectively^54,57,58^. When irradiation is carried out in ethanol, a slight blue shift of magnetite bands is observed compared to the reported data^47,49,50,55,56,59,60^. This could be related to the non-equivalent sites in the wustite (FeO) structure^55^ or the bulk-like character of the material^61^. Raman spectra of particles irradiated in ethanol are additionally affected by other bands at 698 (A_1g_), 480 (E_g_), 366 (T_1g_) cm^−1^. These vibrational modes indicate the presence of oxidized iron due to the structural transformation of magnetite to maghemite (Fig. 6h, red bands). Unfortunately, iron carbide has no Raman active vibrational modes^62^, thus the Raman data support the XRD results but only for iron oxides. Despite this, Raman spectroscopy clearly shows that hematite has been reduced and no α-Fe_2_O_3_ was observed in both irradiated samples. Also, the XPS analysis performed before and after irradiation confirms the reduction of hematite (Supporting information, Fig. S4).

**Figure 7:**
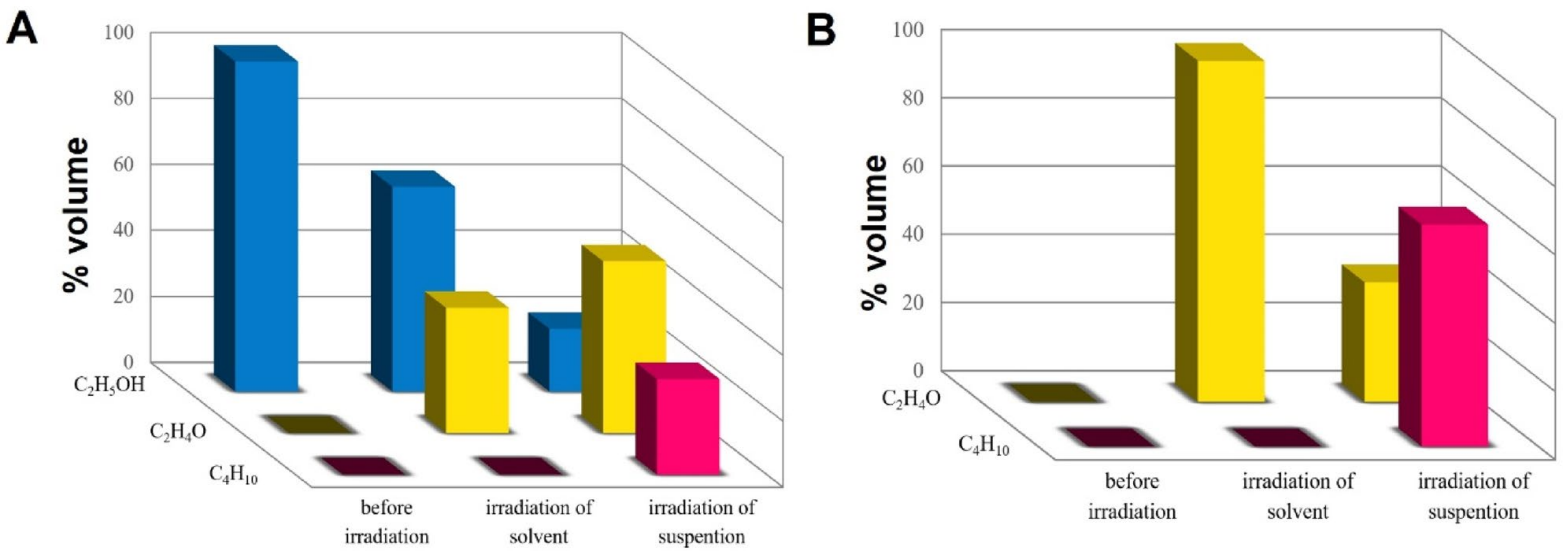
(**A**) The percentage volume composition of the resulting volatile compounds at the outlet of the closed vessel with ethyl acetate; (i) before irradiation, (ii) after three hours of irradiation of the solvent, (iii) after three hours of irradiation of the nanoparticles suspension; (**B**) the percentage composition of the resulting volatile compounds at the outlet of the closed vessel with ethanol; (i) before irradiation, (ii) after three hours of irradiation of the solvent, (iii) after three hours of irradiation of the nanoparticles suspension.

For the GC–MS analysis, gaseous samples at the outlet of the closed vessel with the irradiated liquid were taken in the following cases: (i) control experiment before irradiation, (ii) after three hours of irradiation of the solvent (without nanoparticles), (iii) after three hours of irradiation of the nanoparticles suspension. The same steps were repeated for ethyl acetate and ethanol. The GC–MS analysis for ethyl acetate before irradiation indicates that the outlet constituent contains ethanol (C_2_H_5_OH), ethyl aldehyde (CH_3_CHO), and a trace amount (few ppm) of butane (C_4_H_10_) (Fig. 7A). Analysis of the gaseous products released upon irradiation of the control solvent indicates an increase in the concentration of all identified constituents. The difference was even more prominent when we analyzed the gaseous products when the suspension of nanoparticles was irradiated. In this case, the content of CH_3_CHO, and C_4_H_10_ increased compared to the control sample, while the content of C_2_H_5_OH decreased. No other compounds have been identified.

The GC–MS analysis for ethanol before irradiation indicates that the outlet constituent contains ethyl aldehyde (CH_3_CHO) and butane (C_4_H_10_) (Fig. 7B). There was no detectable ethanol (C_2_H_5_OH). As before, the photolysis of the pure solvent causes an increase in the percentage composition of the resulting volatile compounds compared to the control sample. Upon irradiation of the α-Fe_2_O_3_ NPs suspension, the significant increase in the concentration of both constituents ethyl aldehyde (CH_3_CHO) and butane (C_4_H_10_) was identified compared to the control sample.

The results clearly show solvent photolysis for both ethyl acetate and ethanol. In the case of ethyl acetate, the phenomenon of laser photolysis has been described by other authors^63^, indicating its decomposition into several chemical species, such as CO, CO_2_, CH_4_, C_2_H_4_, C_2_H_6_. In general, photolysis is the reaction of esters of the R_1_COOR_2_ type which successively decompose to form radicals. In the first stage, further molecules and radials are formed: R_1_COO·, ·COOR_2,_ and ·R (alkyl radicals e.g. ·CH_3_, ·C_2_H_5_). Then, radicals of the R_1_COO·, ·COOR_2_ type decompose into CO_2_ and alkyl radicals of the ·R type. Thus, based on the literature data and our results, we can suggest the following reaction pathways during the photolysis of ethyl acetate:

7 $$ {\text{CH}}_{{3}} {\text{COOC}}_{{2}} {\text{H}}_{{5}} + {\text{ h}}\nu \, \to \cdot{\text{CH}}_{{3}} + \, \cdot{\text{COOC}}_{{2}} {\text{H}}_{{5}} $$

8 $$ {\text{CH}}_{{3}} {\text{COOC}}_{{2}} {\text{H}}_{{5}} + {\text{ h}}\nu \to \, \cdot{\text{CH}}_{{3}} {\text{COO }} + \, \cdot{\text{C}}_{{2}} {\text{H}}_{{5}} $$

9 $$ \cdot{\text{COOC}}_{{2}} {\text{H}}_{{5}} \to {\text{ CO}}_{{2}} + \, \cdot{\text{C}}_{{2}} {\text{H}}_{{5}} $$

10 $$ \cdot {\text{C}}_{{2}} {\text{H}}_{{5}} + \, \cdot {\text{C}}_{{2}} {\text{H}}_{{5}} \to {\text{ C}}_{{4}} {\text{H}}_{{{1}0}} . $$

On the other hand, it is well known that during pulsed laser irradiation, the particle temperature exceeds the melting point in a few hundred nanoseconds or less, with heating and cooling rates of 10^11^ K/s and 10^10^ K/s, respectively^44^. In this study, the interval between two consecutive laser pulses is 100 ms (for a pulse repetition rate of 10 Hz)^64^. Thus, it is substantially longer than the time of the successive rapid melting and solidification processes. The liquid phase surrounding the particle acts as a heat-dissipating barrier upon temporary evaporation and as a cooling medium for quenching. The thin layer surrounding the particle could be a layer of very high temperature of gases and/or liquids, which may promote thermal decomposition before the pyrolysis. The results of the GC–MS analysis support this statement. We observe a significant increase in the concentration of volatile products during irradiation of the nanoparticles suspension (Fig. 7A). Therefore, taking into consideration the thermal decomposition, the following reaction pathways for ethyl acetate can be suggested^65^:

11 $$ {\text{CH}}_{{3}} {\text{COOC}}_{{2}} {\text{H}}_{{5}} \to {\text{ 2CH}}_{{3}} {\text{COH}} $$

12 $$ {\text{CH}}_{{3}} {\text{COOC}}_{{2}} {\text{H}}_{{5}} \to {\text{ CH}}_{{3}} {\text{CH}}_{{2}} {\text{OH }} + {\text{ CH}}_{{2}} {\text{CO}} $$

13 $$ {\text{CH}}_{{3}} {\text{COOC}}_{{2}} {\text{H}}_{{5}} \to {\text{ CH}}_{{3}} {\text{COO }}\cdot \, + \, \cdot{\text{ C}}_{{2}} {\text{H}}_{{5}} . $$

Similarly, laser irradiation of ethanol can lead to its photolysis as described elsewhere^66^. The organic liquid decomposes into several chemical species, such as H_2_, CO, C_2_H_4_, C_2_H_6_. If the irradiation is carried out in a mixture of ethanol and hydrogen peroxide, OH radicals may also be present^67^. Ethanol decomposition as a result of a progressive photolysis can lead to the formation of ethyl (∙C_2_H_5_), ethanol (∙CH_3_CHOH), ether (∙CH_3_CHOC_2_H_5_), or acetyl (CH_3_CO∙) radicals^66,67^. As such, based on the literature data and our results, the following reactions of ethanol photolysis can be postulated:

14 $$ {\text{C}}_{{2}} {\text{H}}_{{5}} {\text{OH }} + {\text{ h}}\nu \, \to \, \cdot{\text{C}}_{{2}} {\text{H}}_{{5}} + \, \cdot{\text{OH}} $$

15 $$ {\text{C}}_{{2}} {\text{H}}_{{5}} {\text{OH }} + {\text{h}}\nu \, \to \, \cdot{\text{C}}_{{2}} {\text{H}}_{{5}} {\text{O}} + \, \cdot{\text{H}} $$

16 $$ {\text{C}}_{{2}} {\text{H}}_{{5}} {\text{O}} \cdot \, + {\text{C2H}}_{{5}} {\text{OH }} \to {\text{CH}}_{{3}} {\text{CH}}_{{2}} {\text{OH }} + \, \cdot {\text{CH}}_{{3}} {\text{CHOH}} $$

17 $$ {\text{CH}}_{{3}} {\text{CHOH }} + {\text{ HOCH}}_{{2}} {\text{CH}}_{{3}} + {\text{h}}\nu \, \to \, \cdot {\text{CH}}_{{3}} {\text{CHO}} + {\text{ H}}_{{2}} 0 + \, \cdot {\text{C}}_{{2}} {\text{H}}_{{5}} $$

18 $$ \cdot {\text{C}}_{{2}} {\text{H}}_{{5}} + \, \cdot {\text{C}}_{{2}} {\text{H}}_{{5}} \to {\text{ C}}_{{4}} {\text{H}}_{{{1}0}} $$

19 $$ \cdot{\text{OH }} + \, \cdot{\text{H }} \to {\text{ H}}_{{2}} {\text{O}} $$

20 $$ \cdot{\text{H}} + \, \cdot{\text{H }} \to {\text{ H}}_{{2}} $$

Furthermore, when nanoparticles are dispersed in ethanol, its irradiation can lead to thermal decomposition of the organic liquid as follows^68^:

21 $$ {\text{C}}_{{2}} {\text{H}}_{{5}} {\text{OH}} \to {\text{C}}_{{2}} {\text{H}}_{{5}} + {\text{OH}} $$

22 $$ {\text{C}}_{{2}} {\text{H}}_{{5}} {\text{OH}} \to {\text{ CH}}_{{3}} {\text{CHO }} + {\text{H}}_{{2}} $$

### Mechanism of composite formation

**Figure 8:**
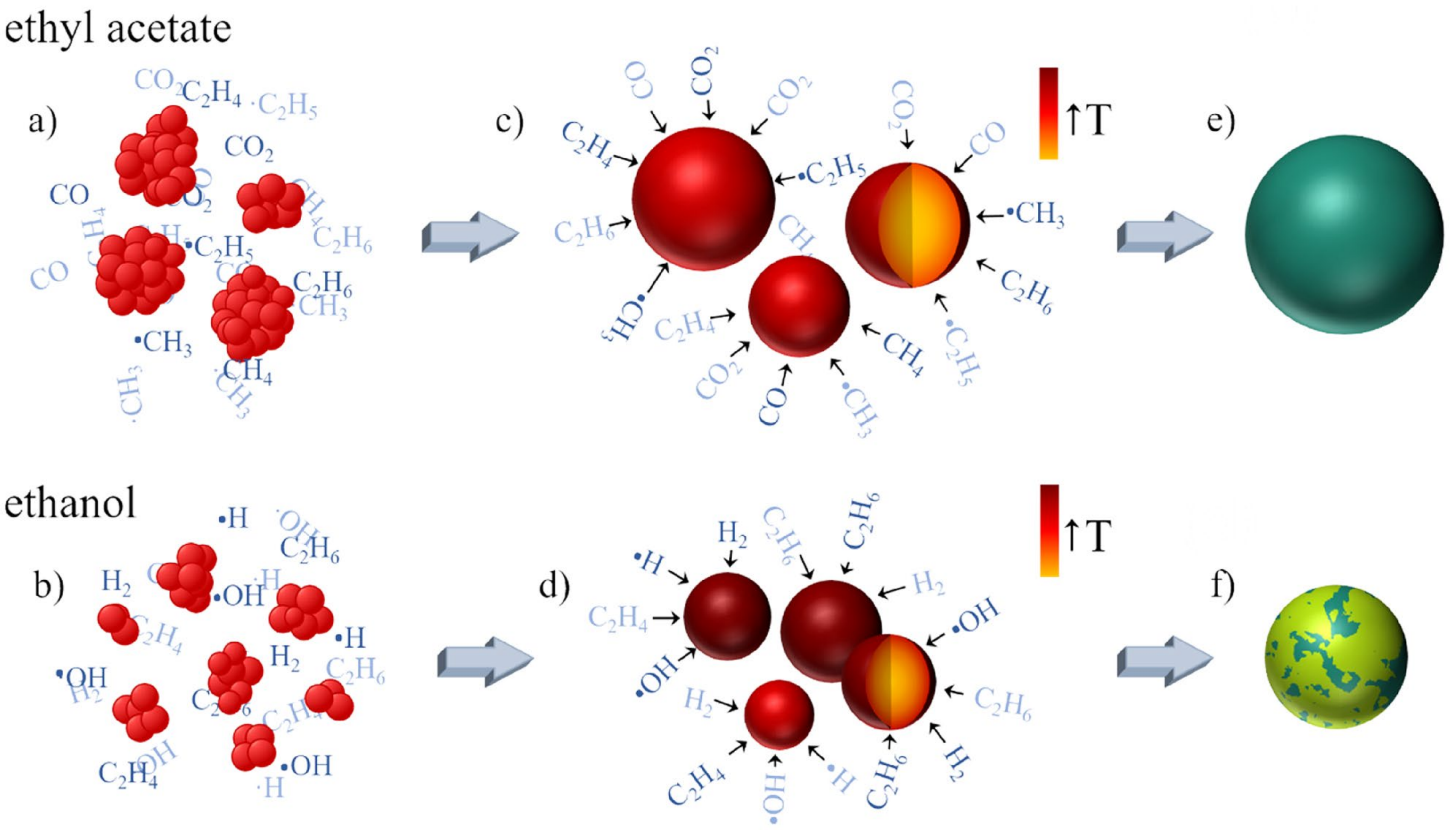
Schematic illustration of morphological and structural evolution of α-Fe_2_O_3_ NPs during laser irradiation in (**a**,**c**,**e**) ethyl acetate and (**b**,** d**,** f**) ethanol; (**a**,**b**) aggregations of α-Fe_2_O_3_ nanoparticles, (**c**,**d**) the temperature distribution in particles during irradiation: particles with a size around 200 nm have the highest temperature (brown), larger or smaller ones, irradiated with the same laser fluence, have lower temperature (dark red); (**e**,**f**) final composite particles; (e) particles obtained in ethyl acetate are composed on magnetite (green), (f) particles obtained in ethanol are more reduced and composed on magnetite (green) and wustite (light green) phases.

Based on the presented numerical and experimental results, we propose a possible mechanism of determining the final size and phase composition of iron oxide particles obtained during laser irradiation. The entire process of particle growth in two different organic liquids is schematically shown in Fig. 8. The first step is the agglomeration of primary particles (Fig. 8a,b). In the liquid with a lower dielectric constant such as ethyl acetate (ε_r_ = 6), most agglomerates have a large diameter (Fig. 8a) (500 nm in this study, Table S1), but with the increase of the dielectric constant (i.e. ethanol, ε_r_ = 24.6), these values shift towards lower diameters (Fig. 8b) (200 nm in this studies, Table S1). In the second step, as a result of the repeated fast-melting and solidification of the agglomerates, spherical submicron-sized particles are formed. However, as large agglomerates are found in ethyl acetate, the composites formed there are larger. Thus, we conclude that the difference in the final size of the composite microsphere is driven by the degree of agglomeration of the primary NPs. The final phase composition of the particles is another issue. According to our calculations, particles with a size around 200 nm will have the highest temperature (Fig. 8c). Larger or smaller ones, irradiated with the same laser fluence, will have lower temperature (Fig. 8d). Numerical calculations indicated that among all the particles present in the liquid environment, those with a diameter of 200 nm would be the first to decompose (assuming a wavelength and the laser fluence of 532 nm and 390 mJ/pulse^.^cm^2^, respectively). Those with larger and smaller diameters require greater laser fluences to maintain the same reduction pathway of iron oxide. It means that the particles synthesized in ethanol can be rapidly reduced to magnetite or wustite (Fig. 8f). On the other hand, assuming the same irradiation conditions in the ethyl acetate environment, as larger sizes are available, the iron oxide reduction progress will be substantially lower (Fig. 8e). The appearance of metallic iron and iron carbide in the structure of the composite must be explained as owed to phenomena other than the thermal decomposition. We suggest that the photolysis of the solvent is an important factor for determining the effectiveness of solvent toward the reduction progress of wustite. The photolysis process takes place from the beginning of irradiation. Moreover, it is highly intensified when nanoparticles are suspended in an organic liquid. Molecules and radicals, such as CO, CO_2_, CH_4_, C_2_H_4_, C_2_H_6_, ·CH_3_, ·C_2_H_5_, H_2_, C_2_H_6_,·OH, H appear in a thin layer around the particle (Fig. 8a–d). Those are good reducing agents that can adsorb on the particle surface and afterward diffuse into its inner layer, causing the primary wustite particles to be converted into metallic iron or iron carbide. However, for larger particles, the rate of reduction becomes limited due to the increasing diffusion pathway^69,70^ and thus, we can assume that the outer surface of the particle will be more reduced than its interior. In conclusion, due to the repeated processes of agglomeration, absorption, decomposition, and melting, during pulsed laser irradiation, spherical submicron particles will form, that are smaller and more reduced in ethanol than in ethyl acetate (Fig. 8e,f).

## Conclusions

To conclude, using morphological and structural characterization techniques, gas chromatography, and theoretical calculations of thermal processes throughout laser melting and solidification, we performed detailed studies on solvent-particles chemical interactions during particles formation by pulsed laser irradiation of α-Fe_2_O_3_. The results showed the influence of the solvent on the size of raw α-Fe_2_O_3_ NPs agglomerates and composite microspheres. The greater the dielectric constant of the organic liquid, the smaller the final size of the formed material. In particular, the hydrodynamic diameter of the agglomerates of raw α-Fe_2_O_3_ NPs varies from 800 to 200 nm, for toluene and acetone suspension, respectively. The average sizes of composite particles obtained by laser irradiation of Fe_2_O_3_ NPs decreases from 600 to 400 nm with an increase in the dielectric constant of the organic liquid as well. The experimental results also suggest that the used solvent and the size of the agglomerates, directs the reduction stages of the starting hematite phase. The reduction of raw α-Fe_2_O_3_ NPs in ethyl acetate ends up with magnetite, while in ethanol, it ends up with iron and iron carbide. The theoretical analysis showed the clear minimum in the laser fluence curves for particles with a diameter of about 200 nm, which indicates that these have higher temperatures than the larger ones. Ignoring the cooling effect, the calculated temperature of 200 nm particles, irradiated with 390 mJ/pulse^.^cm^2^, reaches a maximum value of 2320 K. When considering heat dissipation, the particles suspended in ethanol reach the melting point (1870 K), while those in ethyl acetate slightly exceed it (1902 K). Phase equilibrium diagrams proved the existence of Fe, FeO, and Fe_3_O_4_ as the preferred phases at about 1900 K. The results of the chromatographic analysis confirm the photolysis of the solvent during laser irradiation and explain the reduction of wustite to iron and iron carbide. Our study examining the interaction between organic liquid and α-Fe_2_O_3_ NPs contributes to our understanding of the process of submicron particle formation during the pulsed laser irradiation process.

## Methods

### Submicron particles synthesis

α-Fe_2_O_3_ nanoparticles (α-Fe_2_O_3_ NPs, Sigma Aldrich powder form, average size 20 nm, purity 99.5%, 0.5 mM) were dispersed in liquid medium. The effects of four solvents, namely toluene, ethyl acetate, acetone, and ethanol were investigated. The 5 mL of resulting suspension was ultrasonically mixed and transferred to a sealed cell equipped with quartz window permeable for 523 nm wavelength. Then, the mixture was irradiated with unfocused pulsed laser beam generated by Nd:YAG laser operating in the second harmonic mode at 532 nm wavelength and with 30 Hz repetition rate. Energy density of 180 mJ/pulse^.^cm^2^ was used to irradiate nanoparticles colloids for one hour. During irradiation, an ultrasonic stirring was maintained to prevent sedimentation and gravitational settling of the suspension.

For GC–MS (gas chromatography–mass spectrometer) analysis, the same procedure as in the above section was carried out to synthesize the submicron particles, with the difference that the α-Fe_2_O_3_ nanoparticles (US-NANO, powder form, average size 30 nm, purity 99.5%, 2.5 mM) were dispersed in two solutions, i.e. ethanol and ethyl acetate. 15 ml of the resulting suspension was irradiated by Nd:YAG laser operating in the second harmonic mode at 532 nm wavelength and with 10 Hz repetition rate for three hours, and the laser energy density was 390 mJ/pulse^.^cm^2^.

### Characterizations

The morphology of the obtained particles was observed by scanning electron microscope (Hitachi S4800 and Tescan Vega3). The average particle size was determined by measuring the diameters of 200 particles from each SEM image. The crystal structure of particles was determined with an X-ray diffractometer (XRD, PANalytical X’Pert Pro). The detection was performed using the Cu K_α_ (α = 1.54 Å) radiation at operating current and voltage of 30 mA and 40 kV, respectively. Raman spectra were collected using WITec confocal Raman microscope (CRM alpha 300R) equipped with λ = 532 nm laser and air Olympus MPLAN (100x/0.90NA) objective. Data were accumulated with 60 scans with an integration time of 30 s and a resolution of 3 cm^−1^. The spectrometer monochromator was calibrated using the Raman scattering line of a silicon plate (520.7 cm^−1^). Iron oxides are usually vulnerable to heat-induced phase changes^47,71^, therefore the spectra were collected at very low laser power to avoid the thermal alteration induced by laser (~ 0.45 mW). The baseline correction and cosmic ray removal were carried out using WitecFour Plus software (version 5.3) while the peak fitting analysis was done using GRAMS software package (version 9.2). The X-ray Photoelectron Spectroscopy (XPS) analysis has been performed in a multi-chamber UHV system equipped with a hemispherical analyzer (SES R4000, Gammadata Scienta). The unmonochromatized AlK_α_ (1486.6 eV) X-ray source with the anode operating at 12 kV and 15 mA current emission was applied to generate core excitation. The spectrometer was calibrated according to ISO 15472:2001. The energy resolution of the system operating at a constant pass energy of 100 eV was 0.9 eV (measured as a full width at half maximum for Ag 3d_5/2_ excitation line). The base pressure in the analysis chamber was about 1 × 10^–10^ mbar and about 3 × 10^–9^ mbar during the experiment. The area of sample analysis was about 4 mm^2^ (5 × 0.8 mm).

The binding energy (BE) of adventitious carbon species was used to correct measured spectra for surface charging (C 1 s line at BE = 285.0 eV). Intensities were estimated by calculating the integral of each peak (CasaXPS 2.3.23) after subtraction of the Shirley-type background, and fitting the experimental curves with a combination of Gaussian and Lorentzian lines of variable proportions (70:30). The hydrodynamic diameter of α-Fe_2_O_3_ nanoparticles was examined by dynamic light scattering (DLS) with a Malvern Zetasizer Nano-ZS instrument equipped with a He–Ne laser (λ = 633 nm) and operated at a backscattering angle of 173°.

### GC–MS analysis

**Figure 9:**
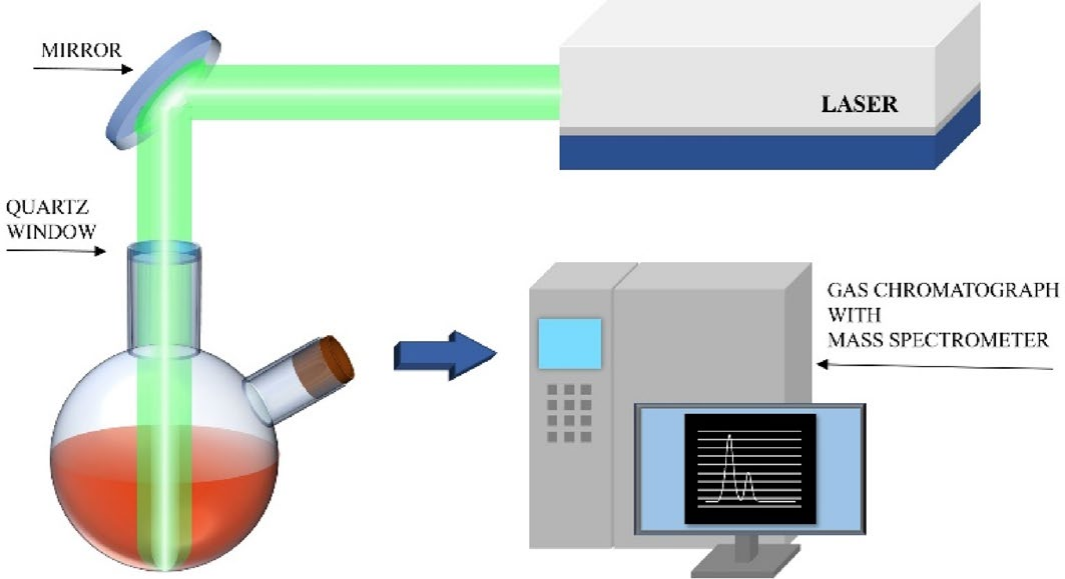
Experimental setup used to analyse gas samples produced during the laser irradiation process.

The composition of the volatile products of gas sample taken during laser irradiation of colloidal suspension was analyzed using gas chromatography (Agilent 6890 N GC) equipped with mass spectrometer (Agilent 5975 MSD). The gas sampling system is shown schematically in Fig. 9. The reaction cell was equipped with glass output located in the upper part of the cell being free from liquid medium. 250 µL of gaseous sample was captured from the cell using glass syringe at three time points: before irradiation (t = 0), after 3 h of irradiation of solvent without nanoparticles and after irradiation of nanoparticles dispersed in solvent (t = 3 h). Next, each sample was diluted to 100 ml using synthetic air 5.5. The diluted gaseous samples were adsorbed on Tenax-TA (poly 2,6-diphenyl-p-phenylene oxide) film located inside glass tube. The chromatographic separation was carried out on a 60 m long poly(dimethylsiloxane) DB-1 column with an internal diameter of 0.32 mm and film thickness of 5 µm. The initial temperature of 60 °C was linearly increased to 120 °C at a rate of 5 °C min^−1^ and then to 230 °C at a rate of 15 °C min^−1^. The final temperature was held for 6 min. A constant column gas flow rate of 40 mL min^−1^ was controlled using a diaphragm pump and was applied throughout the chromatographic run. The mass spectra of compounds were obtained in full scan mode (SCAN) from m/z 32 to 100 amu. For quantitative determination of the analyte, the samples were examined in selected ion monitoring mode (SIM). Retention times and m/z ratios selected for quantitative determination are presented in Table 2. The components were identified by comparison of their mass spectra with library entries (National Institute of Standards and Technology, USA).

**Table 2:** Retention times and characteristic ions selected for quantitative determination in SM mode.

Compound	Chemical formula	Retention time (min)	Mass to charge ratio (m/z)
acetic aldehyde	CH_3_COH	6,45	42, 43, 44
butane	C_4_H_10_	7,37	41, 43, 58
ethanol	C_2_H_5_OH	9,1	31, 45, 46
ethyl acetate	CH_3_COOC_2_H_5_	15,91	43, 45, 61, 70, 73

### Thermodynamic and thermal calculations

Calculations of the thermodynamic equilibrium in F-O-C-H system were conducted using HSC Chemistry package software (ver. 10.x, https://www.hsc-chemistry.com/). Matlab software (ver. R2022a, https://www.mathworks.com/products/new_products/latest_features.html) was used for coding and numerical solution of differential equation of heating–cooling model. Mie Plot software (ver. 4620, http://www.philiplaven.com/mieplot.htm) was used for plotting the particle absorption cross section and absorption efficiency against particle diameter.

## Supplementary Information

Supplementary Information.

## Data Availability

The datasets used and/or analyzed during the current study available from the corresponding author on reasonable request.

## References

[1] Zhang D, Gökce B, Barcikowski S (2017). Laser synthesis and processing of colloids: Fundamentals and applications. Chem. Rev..

[2] Neddersen J, Chumanov G, Cotton TM (1993). Laser-ablation of metals—a new method for preparing sers active colloids. Appl. Spectrosc..

[3] Mafune F, Kohno J, Takeda Y, Kondow T, Sawabe H (2000). Formation and size control of sliver nanoparticles by laser ablationin aqueous solution. J. Phys. Chem. B.

[4] Liang S-X, Salamon S, Zerebecki S, Zhang L-C, Jia Z, Wende H, Reichenberger S, Barcikowski S (2021). A laser-based synthesis route for magnetic metallic glass nanoparticles. Script. Mater..

[5] Lee, I., Hana, S. W., Kim, K. Production of Au–Ag alloy nanoparticles by laser ablation of bulk alloys. *Chem. Commun*. 1782–1783 (2001). 10.1039/b105437f 12240313

[6] Amendola V, Meneghetti M, Bakr OM, Riello P, Polizzi S, Anjum DH, Fiameni S, Arosio P, Orlando T, Fernandez CJ, Pineider F, Sangregorioj C, Lascialfari A (2013). Coexistence of plasmonic and magnetic properties in Au_89_Fe_11_ nanoalloys. Nanoscale.

[7] Guadagnini A, Agnoli S, Badocco D, Pastore P, Pilot R, Ravelle-Chapuis R, Fernández van Raap MB, Amendola V (2021). Kinetically stable nonequilibrium gold-cobalt alloy nanoparticles with magnetic and plasmonic properties obtained by laser ablation in liquid. ChemPhysChem.

[8] Jakobi J, Petersen S, Menéndez-Manjón A, Wagener P, Barcikowski S (2010). Magnetic alloy nanoparticles from laser ablation in cyclopentanone and their embedding into a photoresist. Langmuir.

[9] Liu QX, Wang CX, Zhang W, Wang GW (2003). Immiscible silver–nickel alloying nanorods growth upon pulsed-laser induced liquid/solid interfacial reaction. Chem. Phys. Lett..

[10] Gordon E, Karabulin A, Matyushenko V, Sizov V, Khodos I (2014). Stability and structure of nanowires grown from silver, copper and their alloys by laser ablation into superfluid helium. Phys. Chem. Chem. Phys..

[11] Zhang J, Chen G, Guay D, Chaker M, Ma D (2014). Highly active PtAu alloy nanoparticle catalysts for the reduction of 4-nitrophenol. Nanoscale.

[12] Malviya KD, Chattopadhyay K (2014). Synthesis and mechanism of composition and size dependent morphology selection in nanoparticles of Ag–Cu alloys processed by laser ablation under liquid medium. J. Phys. Chem. C.

[13] Amendola V, Scaramuzza S, Agnoli S, Polizzi S, Meneghetti M (2014). Strong dependence of surface plasmon resonance and surface enhanced Raman scattering on the composition of Au–Fe nanoalloys. Nanoscale.

[14] Johny J, Kamp M, Prymak O, Tymoczko A, Wiedwald U, Rehbock Ch, Schürmann U, Popescu R, Gerthsen D (2021). Formation of Co–Au core-shell nanoparticles with thin gold shells and soft magnetic ε-cobalt cores ruled by thermodynamics and kinetics. J. Phys. Chem. C.

[15] Ishikawa Y, Feng Q, Koshizaki N (2010). Growth fusion of submicron spherical boron carbide particles by repetitive pulsed laser irradiation in liquid media. Appl. Phys. A.

[16] Wang H, Pyatenko A, Kawaguchi K, Li X, Swiatkowska-Warkocka Z, Koshizaki N (2010). Selective pulsed heating for the synthesis of semiconductor and metal submicrometer spheres. Angew. Chem Int. Ed..

[17] Wang HQ, Kawaguchi K, Pyatenko A, Li XY, Swiatkowska-Warkocka Z, Katou Y, Koshizaki N (2012). General bottom-up construction of spherical particles by pulsed laser irradiation of colloidal nanoparticles: A case study on CuO. Chem. Eur. J..

[18] Zhang H, Huang S, Yang X, Yuan R, Chai Y (2021). A SERS biosensor constructed by calcined ZnO substrate with high-efficiency charge transfer for sensitive detection of Pb^2+^. Sens. Actuators, B Chem..

[19] Liu Y, Wang Z, Zhong Y, Tade M, Zhou W, Shao Z (2017). Molecular design of mesoporous NiCo_2_O_4_ and NiCo_2_S_4_ with sub-micrometer-polyhedron architectures for efficient pseudocapacitive energy storage. Adv. Funct. Mater..

[20] Lim H-S, Lee J, Lee S, Kang YS, Sun Y-K, Suh K-D (2017). Walnut-like ZnO@Zn_2_TiO_4_ multicore-shell submicron spheres with a thin carbon layer: Fine synthesis, facile structural control and solar light photocatalytic application. Acta Mater..

[21] Atabaev TS, Lee JH, Han D-W, Hwang Y-H, Kim H-K (2012). Cytotoxicity and cell imaging potentials of submicron color-tunable yttria particles. J Biomed Mater Res Part A.

[22] Pyatenko A, Wang H, Koshizaki N, Tsuji T (2013). Mechanism of pulse laser interaction with colloidal nanoparticles. Laser Photon. Rev..

[23] Tsuji T, Yahata T, Yasutomo M, Igawa K, Tsuji M, Ishikawa Y, Koshizaki N (2013). Preparation and investigation of the formation mechanism of submicron-sized spherical particles of gold using laser ablation and laser irradiation in liquids. Phys. Chem. Chem. Phys..

[24] Tsuji T, Higashi Y, Tsuji M, Ishikawa Y, Koshizaki N (2015). Preparation of submicron-sized spherical particles of gold using laser-induced melting in liquids and low-toxic stabilizing reagent. Appl. Surf. Sci..

[25] Wang HQ, Miyauchi M, Ishikawa Y, Pyatenko A, Koshizaki N, Li Y, Li L, Li XY, Bando Y, Golberg D (2011). Single-crystalline rutile TiO_2_ hollow spheres: Roo-temperature synthesis, tailored visible-light-extinction, and effective scattering layer for quantum dot-sensitized solar cells. J. Am. Chem. Soc..

[26] Wang H, Pyatenko A, Koshizaki N, Moehwald H, Shchukin D (2014). Single-crystalline ZnO spherical particles by pulsed laser irradiation of colloidal nanoparticles for ultraviolet photodetection. ACS Appl. Mater. Interfaces.

[27] Wang HQ, Koshizaki N, Li L, Jia LC, Kawaguchi K, Li XY, Pyatenko A, Swiatkowska-Warkocka Z, Bando Y, Golberg D (2011). Size-tailored ZnO submicrometer spheres: bottom-up construction, size-related optical extinction, and selective aniline trapping. Adv. Mater..

[28] Ishikawa Y, Koshizaki N, Pyatenko A, Saitoh N, Yoshizawa N, Shimizu Y (2016). Nano- and submicrometer-sized spherical particle fabrication using a submicroscopic droplet formed using selective laser heating. J. Phys. Chem. C.

[29] Swiatkowska-Warkocka Z, Kawaguchi K, Wang HQ, Katou Y, Koshizaki N (2011). Controlling exchange bias in Fe_3_O_4_/FeO composite particles prepared by pulsed laser irradiation. Nanoscale Res. Lett..

[30] Swiatkowska-Warkocka Z, Pyatenko A, Shimizu Y, Perzanowski M, Zarzycki A, Jany BR, Marszalek M (2018). Tailoring of magnetic properties of NiO/Ni composite particles fabricated by pulsed laser irradiation. Nanomaterials.

[31] Swiatkowska-Warkocka Z, Koga K, Kawaguchi K, Wang H, Pyatenko A, Koshizaki N (2013). Pulsed laser irradiation of colloidal nanoparticles: A new synthesis route for the production of non-equilibrium bimetallic alloy submicrometer spheres. RSC Adv..

[32] Swiatkowska-Warkocka Z, Pyatenko A, Krok F, Jany BR, Marszalek M (2015). Synthesis of new metastable nanoalloys of immiscible metals with a pulse laser technique. Sci. Rep..

[33] Swiatkowska-Warkocka Z, Pyatenko A, Koga K, Kawaguchi K, Wang H, Koshizaki N (2017). Various morphologies/phases of gold-based nanocomposite particles produced by pulsed laser irradiation in liquid media insight in physical processes involved in particles formation. J. Phys. Chem. C.

[34] Suehara K, Takai R, Ishikawa Y, Koshizaki N, Omura K, Nagata H, Yamauchi Y (2021). Reduction mechanism of transition metal oxide particles in thermally induced nanobubbles during pulsed laser melting in ethanol. ChemPhysChem.

[35] McGrath TE, Diebold GJ, Bartels DM, Crowell RA (2002). Laser-initiated chemical reactions in carbon suspensions. J. Phys. Chem. A.

[36] D’Angelo D, Filice S, Miritello M, Bongiorno C, Fazio E, Neri F, Compagnini G, Scales S (2018). β-Bi_2_O_3_ reduction by laser irradiation in a liquid environment. Phys. Chem. Chem. Phys..

[37] Filice S, Fiorenza R, Reitano R, Scalese S, Sciré S, Fisicaro G, Deretzis I, La Magna A, Bongiorno C, Compagnini G (2020). TiO_2_ colloids laser-treated in ethanol for photocatalytic H_2_ production. ACS Appl. Nano Mater..

[38] Haynes, W. M. Handbook of chemistry and physics, 95th edn. Boca Raton: CRC, 2014–2015.

[39] Verwey EJW, Overbeek JTh (1995). Theory of the stability of lyophobic colloids. J. Colloid Sci..

[40] Liu J, Liang C, Zhu X, Lin Y, Zhang H, Wu Sh (2016). Understanding the solvent molecules induced spontaneous growth of uncapped tellurium nanoparticles. Sci. Rep..

[41] Donner JS, Baffou G, McCloskey D, Quidant R (2011). Plasmon-assisted optofluidics. ACS Nano.

[42] Baukal CE (2000). Heat transfer in industrial combustion.

[43] Plech A, Kotaidis V, Grésillon S, Dahmen C, Von Plessen G (2004). Laser-induced heating and melting of gold nanoparticles studied by time-resolved x-ray scattering. Phys. Rev. B.

[44] Sakaki S, Ikenoue H, Tsuji T, Ishikawa Y, Koshizaki N (2017). Pulse-width dependence of the cooling effecton sub-micrometer ZnO spherical particle formation by pulsed-laser melting in a liquid. ChemPhysChem.

[45] Hashimoto Sh, Werner D, Uwada T (2012). Studies on the interaction of pulsed lasers with plasmonic gold nanoparticles toward light manipulation, heat management, and nanofabrication. J. Photochem. Photobiol. C.

[46] Werner D, Hashimoto Sh (2011). Improved working model for interpreting the excitation wavelength-and fluence-dependent response in pulsed laser-induced size reduction of aqueous gold nanoparticles. J. Phys. Chem. C.

[47] de Faria DLA, Silva SV, de Oliveira MT (1997). Raman microspectroscopy of some iron oxides and oxyhydroxides. J. Raman Spectrosc..

[48] Shebanova ON, Lazor P (2003). Raman spectroscopic study of magnetite (FeFe_2_O_4_): a new assignment for the vibrational spectrum. J. Solid Chem..

[49] Pinna N, Grancharov S, Beato P, Bonville P (2005). Magnetite nanocrystals: nonaqueous synthesis, characterization, and solubility. Chem. Mater..

[50] Maslar JE, Hurst WS, Bowers WJ, Hendricks JH, Aquino MI (2000). In situ Raman spectroscopic investigation of aqueous iron corrosion at elevated temperatures and pressures. J. Electrochem. Soc..

[51] Cesar I, Sivula K, Kay A, Zboril R, Grzel M (2008). Influence of feature size, film thickness, and silicon doping on the performance of nanostructured hematite photoanodes for solar water splitting. J. Phys. Chem. C.

[52] Shim SH, Duffy TS (2002). Raman spectroscopy of Fe_2_O_3_ to 62GPa. Am. Miner..

[53] Mirzaei A, Janghorban K, Hashemi B, Hosseini SR, Bonyani M, Leonardi SG, Bonavita A, Neri G (2016). Synthesis and characterization of mesoporous -Fe_2_O_3_ nanoparticles and investigation of electrical properties of fabricated thick films. Process. Appl. Ceramics.

[54] Chamritski I, Burns G (2005). Infrared-and Raman-active phonons of magnetite, maghemite, and hematite: A computer simulation and spectroscopic study. J. Phys. Chem. B.

[55] Thibeau RJ, Brown CW, Heidersbach RH (1978). Raman spectra of possible corrosion products of iron. Appl. Spectrosc..

[56] Malendres CA, Pankuch M, Li YS, Khnight RL (1992). Surface enhanced Raman spectroelectrochemical studies of the corrosion films on iron and chromium in aqueous solution environments. Electrochim. Acta.

[57] Book raman spectroscopy

[58] Shebanova ON, Lazor P (2003). Raman study of magnetite (Fe_3_O_4_): laser-induced thermal effects and oxidation. J. Raman Spectrosc..

[59] Xi G, Wang C, Wang X (2008). The oriented self-assembly of magnetic Fe3O4 nanoparticles into monodisperse microspheres and their use as substrates in the formation of Fe3O4 nanorods. Eur. J. Inorg. Chem..

[60] Raman RKS, Gleeson B, Young DJ (1998). Laser Raman spectroscopy: A technique for rapid characterisation of oxide scale layers. Mat. Sci. Techn..

[61] Chourpa I, Douziech-Eyrolles L, Ngaboni-Okassa L, Fouquenet J-F, Cohen-Jonathan S (2005). Molecular composition of iron oxide nanoparticles, precursors for magnetic drug targeting, as characterized by confocal Raman microspectroscopy. Analyst.

[62] Park E, Ostrovski O, Zhang J, Thomson S, Howe R (2001). Characterization of phases formed in the iron carbide process by X-Ray diffraction, mossbauer, X-ray photoelectron spectroscopy, and raman spectroscopy analyses. Met. Mater. Trans. B.

[63] Judeikis HS, Siegel S (1965). Photolysis of low-temperature glasses I Ethanol and ether at 77°K. J. Chem. Phys..

[64] Sakaki Sh, Ikenoue H, Tsuji T, Ishikawa Y, Koshizaki N (2018). Influence of pulse frequency on synthesis of nano and submicrometer spherical particles by pulsed laser melting in liquid. Appl. Surf. Sci..

[65] Vargas DC, Salazar S, Mora JR, Van Geem KM, Streitwieser DA (2020). Experimental and theoretical study of the thermal decomposition of ethyl acetate during fast pyrolysis. Chem. Eng. Res. Des..

[66] Johnsen RH (1959). Photolysis of gamma-ray produced free radicals in ethanol at low temperatures. J. Phys. Chem..

[67] Rotzoll G (1985). High-temperature pyrolysis of ethanol. J. Anal. Appl. Pyrol..

[68] Sivaramakrishnan R, Su M-C, Michael JV, Klippenstein SJ, Harding LB, Ruscic B (2010). Rate constants for the thermal decomposition of ethanol and its bimolecular reactions with OH and D: Reflected shock tube and theoretical studies. J. Phys. Chem. A.

[69] Spreitzer D, Schenk J (2019). Reduction of iron oxides with hydrogen—a review. Steel Res. Int..

[70] Jozwiak WK, Kaczmarek E, Maniecki TP, Ignaczak W, Maniukiewicz W (2007). Reduction behavior of iron oxides in hydrogen and carbon monoxide atmospheres. Appl. Catal. A.

[71] Hanesch M (2009). Raman spectroscopy of iron oxides and (oxy)hydroxides at low laser power and possible applications in environmental magnetic studies. Geophys. J. Int..

