# Supplementary material for: Solvent-particles interactions during composite particles formation by pulsed laser melting of α-Fe2O3
Source: Sci Rep. 2022 Jul 13;12:11950. doi: 10.1038/s41598-022-15729-y (PMC9279393; doi:10.1038/s41598-022-15729-y)
Supplement: Supplementary file 1 — Supplementary Information. [file 41598_2022_15729_MOESM1_ESM.docx]

Supporting Information

| **Solvent-particles interactions during composite particles**  **formation by pulsed laser melting of α-Fe_2_O_3_ in liquids** |
| --- |
| M.S. Shakeri^1^, O. Polit^1^, B. Grabowska-Polanowska^2^, A. Pyatenko^3^, K. Suchanek^4^, M. Dulski^5^, J.Gurgul^6^,  Z. Swiatkowska-Warkocka^1*^  *^1^ Institute of Nuclear Physics Polish Academy of Sciences, PL-31342 Krakow, Poland*  *^2^ Institute of Technology and Life Sciences, Ułanów 21B, 31-450 Kraków, Poland*  *^3^ The National Institute of Advanced Industrial Science and Technology (AIST), Tsukuba, Ibaraki 305-8560, Japan*  *^4^ Department of Physics, Cracow University of Technology, Podchorążych 1, 30-084 Kraków, Poland*  *^5^ University of Silesia, 40-007 Katowice, Poland*  *^6^ Jerzy Haber Institute of Catalysis and Surface Chemistry Polish Academy of Sciences, Niezapominajek 8,*  *30-239 Krakow, Poland* |

**Table S1** Size distribution of α-Fe_2_O_3_ agglomerates in solvents with different dielectric constant measured with DLS .

| organic solvent | dielectric constant | agglomerates diameter [nm] |
| --- | --- | --- |
| toluene | 2.4 | 800 |
| ethyl acetate | 6 | 506 |
| acetone | 20.7 | 240 |
| ethanol | 24.6 | 202 |


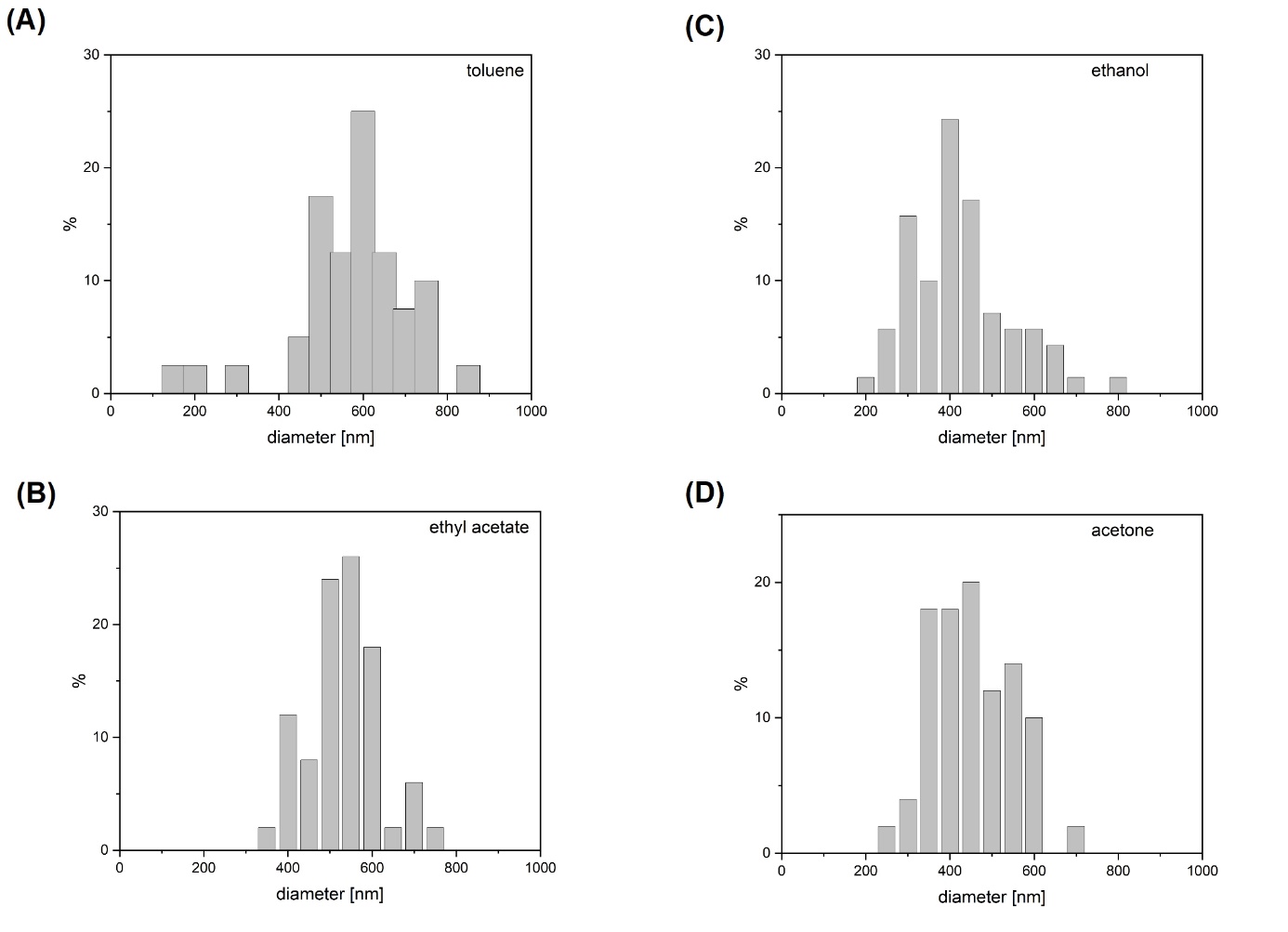


**(E)**


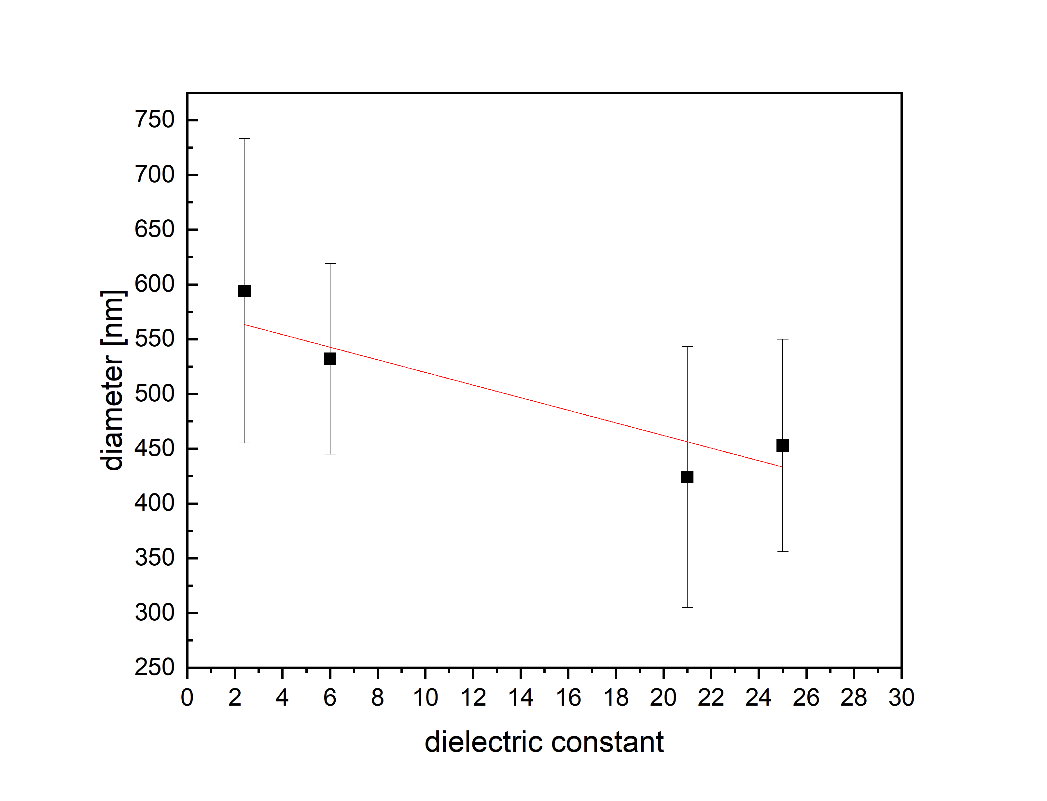


**Figure S1** Size distribution of particles obtained after laser irradiation (532nm, 390 mJ/pulse^.^cm^2^, 1h) in various solvents, (A) toluene, (B), ethyl acetate, (C) ethanol, (D) acetone (from samples analyzed in Figure 1), and dielectric constant dependence of the mean size (with standard deviation) of obtained particles (E).


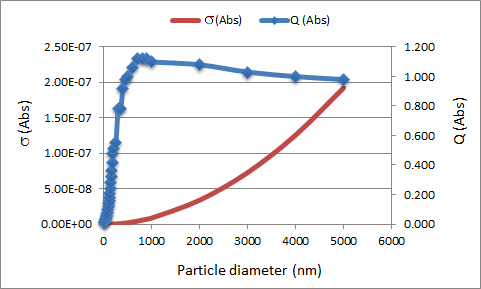


**Figure S2** Absorption cross section and absorption efficiency of spherical Fe_2_O_3_ particles irradiated with wavelength of 532 nm calculated using Mie theory.

**Table S2** The thermodynamic constants for ethanol and ethyl acetate.

| Thermodynamic values | T_0_ (K) | K_l_ (Wm^-2^K^-1^) | Nu_d_ |
| --- | --- | --- | --- |
| Ethanol | 298 | 0.171 | 2 |
| Ethyl Acetate | 298 | 0.137 | 2 |


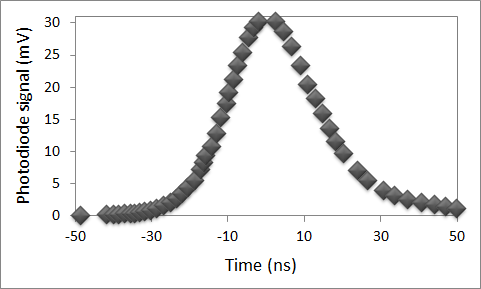
**(A)**

**(B)**

*
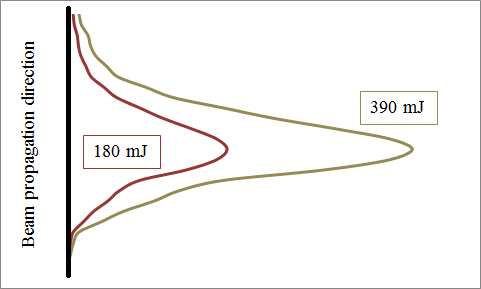
*

**Figure S3** (A) The temporal profile of the pulse of Nd:YAG laser with 10 ns time duration and (B) the intensity profile of the laser for fluence 180 mJ and 390 mJ.


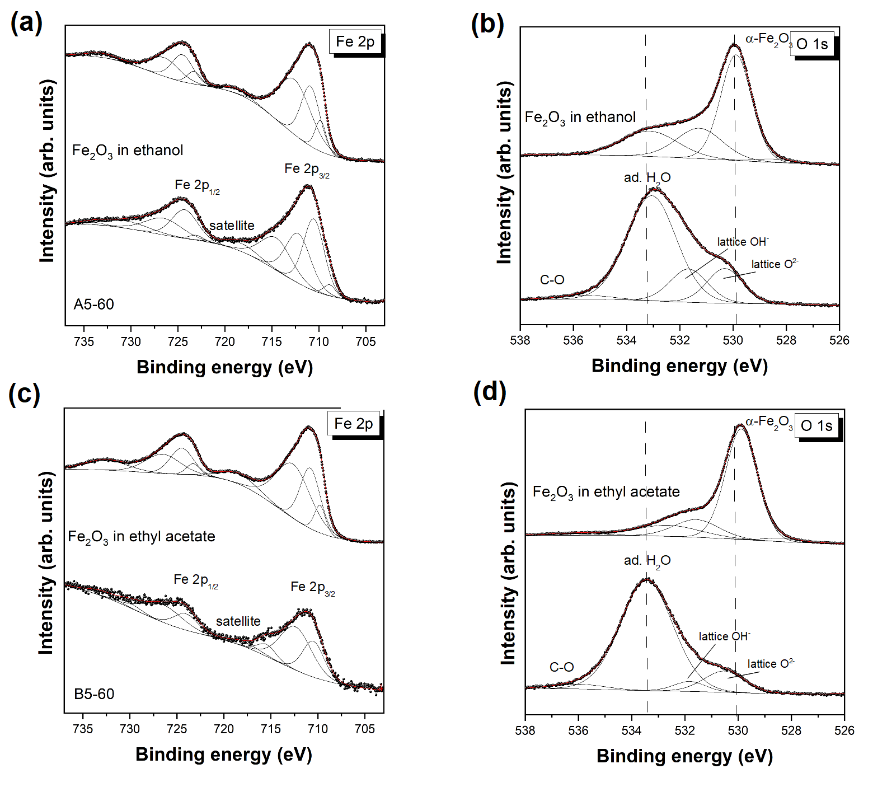


**Figure S4** XPS results for α-Fe_2_O_3_ in ethanol (top) and ethyl acetate (down) before and after laser irradiation (532nm, 390 mJ/pulse^.^cm^2^, 1h). Fe 2p XPS spectrum of particles in ethanol (a) and ethyl acetate (c), O 1s XPS spectrum of particles in ethanol (b) and ethyl acetate (d). Dash lines are guide to the eye.

The BE region of 700-740 eV shown in Fig. S3 reveals very complex spectral features of the Fe 2p photoelectron spectra. The Fe 2p core levels are split into 2p_3/2_ and 2p_1/2_ doublets due to the spin-orbit coupling, whereas the multiplet splitting effect is responsible for the presence of satellites and the broadening of the peaks. It is worth noting, that strong shake-up satellites of varying intensities can be observed only for high-spin compounds [1]. Both, the Fe 2p_3/2_ and Fe 2p_1/2_ peak positions and the value of doublet splitting are sensitive to the oxidation state of iron, as well as its coordination [2]. However, it is rather difficult to quantify relative distribution of Fe^2+^ and Fe^3+^ species due to the inability to accurately determine the true baseline [3].

To describe obtained Fe 2p spectra properly, it was necessary to use two or three doublets and corresponding satellites. The satellites are rather distinguishable, unfortunately they overlap energetically the main photoelectron Fe 2p lines, which makes the analysis much more challenging. In such cases, the binding energy values of Fe 2p peaks are not sufficient and it is necessary to refer to the reference XPS spectra of pure oxides obtained by other authors and to our XRD data as well as Raman spectra. Moreover, detailed analysis of O 1s spectra allows for better identification of the species present, especially in a mix of Fe(II) and Fe(III) species [1].

The Fe 2p spectra of α-Fe_2_O_3_ prepared in ethanol and ethyl acetate are shown in Figs. S3a,c respectively. The main peak with a core-level binding energy of 710.9 eV, accompanied by much smaller peak at 709.8 eV, and together with a shake-up feature at 718.8 eV are fingerprints of α-Fe_2_O_3_ indeed. Both spectra are almost identical to the reference α-Fe_2_O_3_ spectra presented by Kurtz and Henrich [6]. The corresponding O 1s spectra (Figs. S3 b,d show a core-level binding energy of 529.9 eV, which is in line with literature [7,8]. At high binding energy there is a shoulder corresponding to OH groups (531.3 eV) and adsorbed water (533.1 eV). These spectral components are slightly larger for α-Fe_2_O_3_ prepared in ethanol.

Three different contributions of photoelectrons are visible in the Fe 2p spectra of the sample irradiated in ethanol (Fig. S 3a). They can be assigned to Fe^2+^ (708.4 eV), Fe^3+^ (710.5 eV) and FeOOH (712.1 eV) [1,4,5,9]. The O 1s spectrum of sample irradiated in ethanol (Fig. S 3b) show the positions of lattice O^2-^ with BE of 530.3 eV, lattice OH^-^ at 531.5 eV, adsorbed water at 533.4 eV. Small peak with BE ~ 536 eV indicated the formation of organic C–O bonds. Moreover, the ratio of signal intensities from hydroxyl and oxide oxygen is close to 1, in good agreement with the bulk FeOOH stoichiometry [4,10]. It is worth mentioning, that very similar spectra of α-FeOOH [10] and γ-FeOOH [11] are reported in literature, whereas O 1s spectrum of β-FeOOH is quite different [12].

The O 1s spectrum of the sample irradiated in ethyl acetate resembles a goethite spectrum found in sample irradiated in ethanol due to a huge contribution of adsorbed water (Fig. S 3d). On the other hand, the oxide to hydroxide ratio is far from 1, the value expected for the stoichiometric FeOOH. Thus, some other iron phases must be present in studied sample. In the Fe 2p spectrum one can find two main components at 710.5 and 712.4 eV, almost negligible satellite at BE ~ 719 eV and well visible shoulder at BE ~ 716 eV (Fig. S 3c). The Fe 2p_3/2_ core level associated with Fe_3_O_4_ was reported previously at 710.3 eV and did not exhibit any shake-up satellites [7,11]. The absence of these features close to 719 eV is considered to be a fingerprint of Fe_3_O_4_, whereas satellite at 716 eV can be attributed to an Fe^2+^ shake-up [7]. These observations lead us to the conclusion that sample irradiated in ethyl acetate is a mix of goethite and magnetite phases.

Finally, it is worth mentioning that the C 1s spectral lines show the existence of a small number of carbides on the surface of the studied samples. These carbides, especially Fe_3_C, should be visible in the iron XPS spectra at BE of Fe 2p_3/2_ around 708 eV [8], however, the lack of monochromatized X-ray source and the proximity of the lines coming from other Fe^2+^ species do not allow their unambiguous extraction.

**References**

M. C. Biesinger, B. P. Payne, A. P. Grosvenor, L. W. M. Lau, A. R. Gerson, R. St. C. Smart, Appl. Surf. Sci. 257 (2011) 2717-2730.

M. Descostes, F. Mercier, N. Thromat, C. Beaucaire, M. Gautier-Soyer, Appl. Surf. Sci. 165 (2000) 288–302.

D. D. Hawn, B. M. DeKoven, Surf. Interface Anal. 10 (1987) 63-74.

N. S. McIntyre, D. G. Zetaruk, Anal. Chem. 49 (1977) 1521-1529.

P. Grosvenor, B. A. Kobe, M. C. Biesinger, N .S. McIntyre, Surf. Interface Anal. 36 (2004) 1564-1574.

R. L. Kurtz, V. E. Henrich, Surf. Sci. 129 (1983) 345-354.

J. S. Corneille, J.-W. He, D. W. Goodman, Surf. Sci. 338 (1995) 211-224.

C. R. Brundle, T. J. Chuang, K. Wandelt, Surf. Sci. 68 (1977) 459-468.

M. F. Montemor, A. M. P. Simoes, M. G. S. Ferreira, Corrosion 54 (1998) 347-353.

H. Abdel-Samad, P. R. Watson, Appl. Surf. Sci. 108 (1997) 371-377.

L. Martinez, D. Leinen, F. Martin, M. Gabas, J. R. Ramos-Barrado, E. Quagliata, E. A. Dalchiele, J. Electrochem. Soc. 154 (2007) D126-D133.

T. Yang, L. Meng, S. Han, J. Hou, S. Wang, X. Wang, RSC Adv. 7 (2017) 34687–34693.

1. P. Ghods, O. B. Isgor, J. R. Brown, F. Bensebaa, D. Kingston, Appl. Surf. Sci. 257 (2011) 4669-4677.
